# Effectiveness of multidisciplinary team case management: difference-in-differences analysis

**DOI:** 10.1136/bmjopen-2015-010468

**Published:** 2016-04-15

**Authors:** Jonathan Stokes, Søren Rud Kristensen, Kath Checkland, Peter Bower

**Affiliations:** 1 NIHR Greater Manchester Primary Care Patient Safety Translational Research Centre, Manchester Academic Health Science Centre, University of Manchester, Manchester, UK; 2 Manchester Centre for Health Economics, University of Manchester, Manchester, UK; 3 NIHR School for Primary Care Research, Centre for Primary Care, Manchester Academic Health Science Centre, University of Manchester, Manchester, UK

**Keywords:** case management, difference-in-differences, integrated care, multidisciplinary team

## Abstract

**Objectives:**

To evaluate a multidisciplinary team (MDT) case management intervention, at the individual (direct effects of intervention) and practice levels (potential spillover effects).

**Design:**

Difference-in-differences design with multiple intervention start dates, analysing hospital admissions data. In secondary analyses, we stratified individual-level results by risk score.

**Setting:**

Single clinical commissioning group (CCG) in the UK's National Health Service (NHS).

**Participants:**

At the individual level, we matched 2049 intervention patients using propensity scoring one-to-one with control patients. At the practice level, 30 practices were compared using a natural experiment through staged implementation.

**Intervention:**

Practice Integrated Care Teams (PICTs), using MDT case management of high-risk patients together with a summary record of care versus usual care.

**Direct and indirect outcome measures:**

Primary measures of intervention effects were accident and emergency (A&E) visits; inpatient non-elective stays, 30-day re-admissions; inpatient elective stays; outpatient visits; and admissions for ambulatory care sensitive conditions. Secondary measures included inpatient length of stay; total cost of secondary care services; and patient satisfaction (at the practice level only).

**Results:**

At the individual level, we found slight, clinically trivial increases in inpatient non-elective admissions (+0.01 admissions per patient per month; 95% CI 0.00 to 0.01. Effect size (ES): 0.02) and 30-day re-admissions (+0.00; 0.00 to 0.01. ES: 0.03). We found no indication that highest risk patients benefitted more from the intervention. At the practice level, we found a small decrease in inpatient non-elective admissions (−0.63 admissions per 1000 patients per month; −1.17 to −0.09. ES: −0.24). However, this result did not withstand a robustness check; the estimate may have absorbed some differences in underlying practice trends.

**Conclusions:**

The intervention does not meet its primary aim, and the clinical significance and cost-effectiveness of these small practice-level effects is debatable. There is an ongoing need to develop effective ways to reduce unnecessary attendances in secondary care for the high-risk population.

**Strengths and limitations of this study**

- This study addresses a number of shortcomings found in related literature from a recent systematic review.
- The difference-in-differences methods can provide a rigorous assessment under certain conditions while evaluating an intervention in a real-world setting.
- Results are analysed and presented at two levels to show direct effects of the intervention, as well as wider spillover effects of integrated care.
- At the practice level, there may be some selection bias due to voluntary recruitment, although we predict this to be minimal based on our robustness checks.
- At the individual level, results may be prone to some bias in favour of control participants due to the ongoing recruitment strategy versus a single time point propensity matching. Again, we predict this to be minimal, as participants and controls were well matched at the first start date.

## Introduction

An ageing population with increasing number of long-term conditions (LTCs) and complex multimorbidity^1^ ^2^ has caused policymakers to rethink delivery of care.^3^

There is increasing focus on the benefits of ‘integrated care’, to enable a more efficient and effective response to LTCs.^3^ ^4^ There is no consensus definition of what constitutes ‘integrated care’,^5^ and the concept describes many different changes to the health system that can occur at multiple levels.^6^ ^7^ ^8^ Practical implementation examples of integrated care include pooling of funds, joint commissioning, colocation of services, shared clinical records, and at the interface of the health system with the patient (ie, service delivery level) multidisciplinary team (MDT) working and case management.^7^ ^8^

In the UK's National Health Service (NHS), a common model of integrated care is the use of ‘multi-disciplinary team (MDT) case management of high-risk patients’.^9^ ^10^

We undertook a systematic review of this model of integrated care and found few effects across a number of relevant outcomes, barring a small effect on patient satisfaction, and short-term changes in self-reported health status.^6^

We also identified gaps in the current literature. In the review, 78% of included studies were randomised controlled trials (RCTs).^6^ We suggested a complementary role for rigorous quasi-experiments in routine settings to better balance internal and external validity.^11^ ^12^

The majority of studies also measured only direct (individual-level) effects. MDT case management used to manage a subset of patients could lead to broader changes, such as better ‘professional integration’ through team working.^13^ ^14^ These broader changes could lead to effects on the wider patient population, beyond those patients specifically managed by the MDT (what we call ‘spillover effects’).

Our contribution to the evidence base for MDT case management thus involved an evaluation of a local integrated care intervention using a robust quasi-experimental study design. We model effects using two distinct analyses: (1) individual-level analysis (to capture direct effects of the intervention) and (2) practice-level analysis (to capture any potential spillover effects).

### 

#### The intervention

In Central Manchester, the MDT case management is achieved through Practice Integrated Care Teams (PICTs) introduced by the clinical commissioning group (CCG). PICTs conduct case finding, assess the needs of the individual identified, prepare individualised care plans, co-ordinate care and conduct regular review, monitoring and adaptation of the care plan.^15^ The aim of the intervention was to reduce unnecessary attendances in secondary care for the high-risk population.^16^

Table 1 gives an overview of the key aspects of the intervention. Compared with our previous systematic review of similar interventions, it is fairly common, where we identified the majority (58%) employing MDT case management (as opposed to a single case manager), and a predictive risk model as the primary method of identifying suitable patients.^6^ Less commonly, this intervention took place in a system ranked as delivering ‘high’ strength of primary care (ie, strength of primary healthcare orientation of the health system as classified by Starfield and Shi^17^—the majority in the review came from a ‘low’-strength system, eg, USA: 64%). Additionally, the PICT intervention included involvement of a social worker (33% of studies involved a social worker in our previous review), providing further potential for ‘horizontal integration’ (ie, integration between separate organisations at the same level of the health system).^3^

**Table 1:** Overview of intervention

Name of model	Intensity of intervention (patient contacts)	Risk assessment tool (judgement/threshold/predictive risk modelling)	MDT composition	Primary location of intervention	24-hour availability of case manager?	Case load per MDT	Training received by case manager/MDT?	Case management reimbursement method?	IT linkage?
Practice Integrated Care Team (PICT)	Determined case by case—for some weekly, some less frequently	Predictive risk modelling /judgement Combined Predictive Risk Model/clinical judgement	GP, practice nurse, district nurse, social worker and active case manager (with access to other specialists outside of the core team)	GP practice/telephone/home visits—location of patient contacts depended on health professional	No—Health professionals available at their core hours. Emergency routes to community services through the night	Stipulated 2% of adult case load per practice	Yes—Action learning sessions, mock MDTs, initial adopters spreading learning	Payment received as part of wider quality process scheme contract	‘Graphnet’—holds summary care record accessible by multiple providers (read, but not write access). Care plan can be edited by all

GP, general practitioner; MDT, multidisciplinary team.

## Methods

Our study used a quasi-experimental pre–post design with a suitable control group to examine any change in outcomes induced by a policy change intervention—an adapted version of difference-in-differences (DD) analysis.^18^

**Figure 1:**
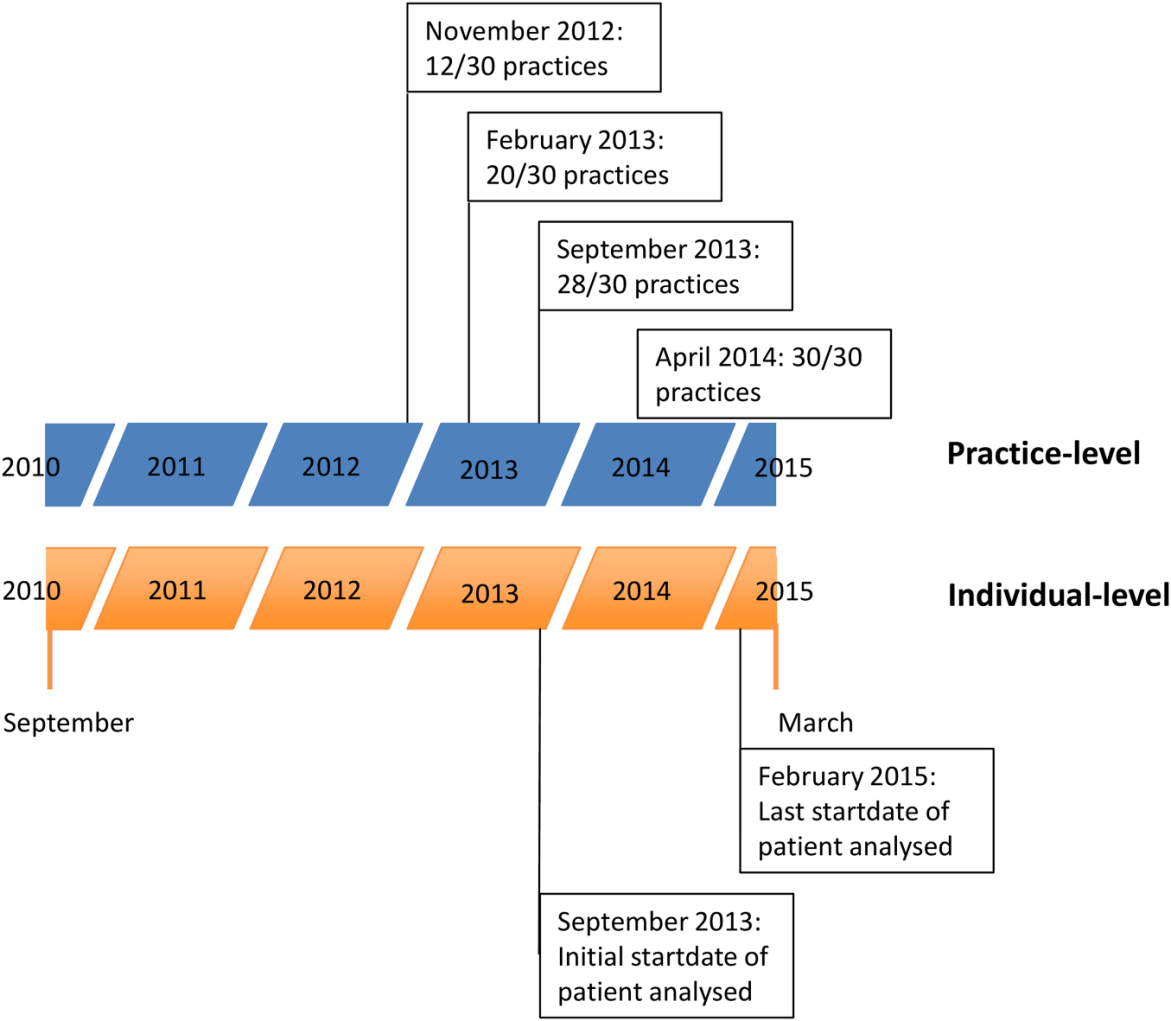
Timeline of analyses highlighting key dates of practices and individual patients included in analysis joining the intervention.

We prepared and analysed data at two distinct levels, each described separately below. Owing to a data governance issue at the CCG, intervention patients could not be identified at the individual level until nearly all practices implemented the intervention (patients were not consented prior to this date, so those joining before could not be included in the analysis—they were also excluded from our control group, so no contamination occurred). Figure 1 summarises the period of analysis for the individual and practice levels, showing the analysis and ‘pretrend’ period (ie, period prior to any practice/individual joining the intervention group) for each.

With the PICT intervention having no single start date, we adapted our analysis to allow for this staged introduction (using a time fixed effect instead of the usual binary post dummy—see equations in practice level and individual level sections in the online supplementary material appendices).^18^ ^19^ The main difference from the standard DD approach is that the intervention and control groups are not static over time, allowing intervention patients/practices to join gradually over the monthly panel datasets, and comparing appropriately at each time point. This method has been used previously in the literature,^19^ ^20^ and we have adapted it to suit data at both of our levels of analyses (explained below, and in more detail in the online supplementary material appendices).

10.1136/bmjopen-2015-010468.supp1 Supplementary data

We analysed anonymised data held by the CCG, from the ‘admitted patient care commissioning dataset’, submitted by all providers in England via the Secondary Uses Service (SUS). The dataset included all patient contacts with secondary care services, demographic data, as well as costs calculated through the national payment by results (PbR, together with local prices for local providers where applicable). For the analysis of pseudonymised/anonymised data, no formal ethics process was deemed necessary. The CCG had themselves previously consented the intervention individuals for use of their data for evaluation purposes. For patient satisfaction at the practice level, we used data from the GP Patient Survey (GPPS—see online supplementary material appendices).

### Data preparation and analysis

All data preparation and analysis was carried out using STATA (V.13) (StataCorp. *Stata Statistical Software: Release 13*. College Station, TX: StataCorp LP., 2013). The DD analysis estimate is unbiased only under the key assumption that the average difference between intervention and control units' trends would be the same in the absence of ‘treatment’ (ie, the PICT intervention).^18^ This ‘parallel trends’ assumption is key to DD analysis and was tested graphically and statistically for each outcome assessed, at each analysis level (see online supplementary material appendices for graphs).

We analysed data distinctly at two levels:

1. Individual level: primary analysis

At the individual level, to obtain parallel pretrends, it was necessary to propensity match intervention patients to controls from within the same CCG (we matched on the characteristics for which the patients were recruited in practice to maximise comparability—see online supplementary material appendices for details). We then analysed 2049 intervention patients versus 2049 matched controls using the best-fitting count model for each outcome.^21^ Outcome measures were summed to a count per patient per month over the period September 2010–March 2015 inclusive, to allow a 3-year pretrend period.

In all models, we adjusted for relevant individual covariates from the directed acyclic graph (DAG—see online supplementary material appendices),^22^ as well as practice fixed effects (to control for any effects caused by characteristics of a specific practice rather than the intervention itself).^23^ We cluster our SEs by practice to deal with concerns of serial correlation.^24^ We took the average partial effect of results (for β_2_*—*see online supplementary material appendices for equation) and report these below (ie, the covariates adjusted absolute change in counts per patient per month). We additionally report the effect size (ES; standardised mean difference) as a measure of practical significance of each result.^25^ We adopted Cohen's rule of thumb for interpreting ESs, that is, 0.2 indicates a small effect, 0.5 a medium and 0.8 a large effect.^26^

#### Stratification by risk score

Patients were recruited to the intervention via risk tool score and clinical judgement. To test whether the highest risk patients (according to risk tool score) benefitted more from the PICT intervention than those with lower calculated risks also treated, we generated a ‘high-risk’ dummy. We reran the individual-level analysis with a difference-in-difference-in-differences (DDD) analysis, using an additional interaction term to determine subgroup effects (see online supplementary material appendices for equation).

- 2. Practice level: secondary analysis of spillover effects

At the practice level, practices gradually took up the intervention over a period of 18 months. At each time point (updating monthly in our dataset), the time fixed effects compare all intervention practices with all ‘controls’ (ie, all those practices that have not yet adopted the intervention, even though they will later adopt the intervention).^19^ ^20^ Outcomes were summed to a count per 1000 patients per month for each of the practices and analysed over the period September 2010–March 2015 inclusive, to overlap with the individual-level analysis.

We used a linear regression model, adjusting for fixed effects for each practice and time period (monthly—see online supplementary material appendices for equation). We cluster our SEs by practice to deal with concerns of serial correlation.^24^

#### Outcome measures

Primary outcome measures for both analyses included:

- Inpatient non-elective admissions
- Re-admissions (30 days)
- Inpatient elective admissions
- Accident and emergency (A&E) visits
- Outpatient visits
- Admissions for ambulatory care sensitive conditions (ACSCs, which we used as a measure of patient safety in a health system with universal health coverage—see online supplementary material appendices for details)

Secondary outcome measures included:

- Total cost of secondary care services (£)
- Length of stay (inpatient)
- Patient satisfaction (practice level only: measured through the GPPS—see online supplementary material appendices)
  General satisfaction
  LTC-specific satisfaction

##### Robustness check

At both levels of analysis, we additionally added a robustness check including a practice-specific time trend. This allows intervention and control practices to follow different trends and can help reveal any indication of the observed effect having absorbed any differences in underlying practice time trends.^18^

At the practice level only, due to the voluntary roll-out of the intervention, we attempted to assess the effects of selection bias using a logistic regression model (including % males; % over 65; practice list size; number of general practitioners (GPs) per thousand patients; total Index of Multiple Deprivation (IMD) score 2010; and total % Quality and Outcomes Framework (QOF) achievement score).^27^ We additionally reran the practice-level analysis excluding those practices recruited to the intervention in wave 1, assuming these to be the practices at most risk of selection bias if it did indeed occur.^19^

## Results

### Individual-level analysis

#### Sample characteristics

A total of 2049 intervention patients were propensity score matched to non-intervention patients from the same CCG. As expected, the differences were small between matched patient baseline characteristics (see table 2).

**Table 2:** Individual baseline characteristics (before and after matching)

	Before matching			After matching		
Mean (unless otherwise indicated)	PICT (SD)	Controls (SD)	SMD	PICT (SD)	Controls (SD)	SMD
N	2049	93 532		2049	2049	
Male (%)	44.3	47.4		44.3	44.1	
Age	67.2 (17.8)	35.3 (22.2)	−1.44	67.2 (17.8)	65.8 (18.7)	−0.07
IMD 2010	40.2 (14.8)	40.6 (16.0)	0.03	40.2 (14.8)	40.2 (15.8)	0.00
MM count baseline	2.7 (2.1)	0.7 (1.2)	−1.63	2.7 (2.1)	2.4 (2.2)	−0.12
Previous inpatient admissions	1.3 (2.1)	0.3 (1.1)	−0.88	1.3 (2.1)	1.2 (2.2)	−0.05
Previous outpatient visits	7.0 (9.6)	1.9 (4.3)	−1.14	7.0 (9.6)	7.2 (9.8)	0.02
Previous A&E visits	1.4 (2.4)	0.5 (1.2)	−0.73	1.4 (2.4)	1.4 (2.5)	0.00

Previous admissions calculated for period 31 August 2012–1 September 2013 (12 months prior to the first intervention patient start date).

A&E, accident and emergency; IMD, Index of Multiple Deprivation; PICT, Practice Integrated Care Team; SMD, standardised mean difference.

Table 3 shows the crude absolute differences in mean outcome measures (PICT patients vs matched controls). As for the DD results in the section below, a negative estimate indicates a relative decrease in admissions for PICT patients compared with controls (ie, a negative intervention effect favours the intervention).

**Table 3:** Average absolute outcomes per patient per month by PICT, preintervention/postintervention

Outcome	Mean (per patient per month)		Difference	Unadjusted intervention effect (difference per patient per month)
	Pre	Post		
*Primary outcomes*				
Inpatient non-electives				
Controls	0.0362	0.0422	0.006	
PICT	0.0550	0.0704	0.0154	0.0094
Inpatient electives				
Controls	0.0365	0.0369	0.0004	
PICT	0.0438	0.0451	0.0013	0.0009
Outpatient admissions				
Controls	0.5019	0.5611	0.0592	
PICT	0.5701	0.7188	0.1487	0.0895
A&E visits				
Controls	0.0808	0.0805	−0.0003	
PICT	0.1061	0.1217	0.0156	0.0159
ACSCs				
Controls	0.0059	0.0078	0.0019	
PICT	0.0093	0.0124	0.0031	0.0012
Re-admissions (30 days)				
Controls	0.0069	0.0082	0.0013	
PICT	0.0115	0.0191	0.0076	0.0063
*Secondary outcomes*				
Total cost of secondary care services (£)				
Controls	168.8746	195.1289	26.2543	
PICT	215.0091	276.9591	61.9500	35.6957
Length of stay (days)				
Controls	0.3943	0.4888	0.0945	
PICT	0.5624	0.7903	0.2279	0.1334

A&E, accident and emergency; ACSCs, ambulatory care sensitive conditions; PICT, Practice Integrated Care Team.

#### DD parallel pretrends

We identified evidence of a significant difference between pretrends for outpatient visits at the individual level. This variable was potentially biased towards a result favouring the PICT intervention over controls. However, we found no statistically significant result favouring either group. All other variables satisfied the parallel trends assumption, with no indication of bias.

#### DD results

Table 4 shows the DD analysis results at the individual level. After adjustment for age, cumulative multimorbidity, IMD domains (excluding health) and practice and time fixed effects, we found a slight increase in inpatient non-elective admissions (0.0053 per patient per month; 95% CI 0.0004 to 0.0102) and 30-day inpatient readmissions (0.0041; 0.0018 to 0.0064). The ESs (0.02 and 0.03) were small.^26^

**Table 4:** Individual-level adjusted model results

Outcome	Adjusted* intervention effect (95% CI) (difference per patient per month)	Effect size†
	Count (NBREG) model	
Primary outcomes		
Inpatient non-electives	**0.0053 (0.0004 to 0.0102)**‡	0.02
Inpatient electives	−0.0011 (−0.0092 to 0.0070)	−0.00
Outpatient visits	0.0399 (−0.0068 to 0.0866)	0.03
A&E visits	0.0103 (−0.0001 to 0.0207)	0.03
ACSCs	0.0001 (−0.0017 to 0.0020)	0.00
Re-admissions (30 days)	**0.0041 (0.0018 to 0.0064)**‡	0.03
Secondary outcomes		
Total cost of secondary care services (£)§	8.1687 (−16.0021 to 32.3396)	0.01
Length of stay (days)	0.0528 (−0.1094 to 0.2151)	0.01

N=224 898 observations; 4098 individuals (period September 2010–March 2015).

bold: withstands practice×time robustness check.

*Adjusted for age, cumulative multimorbidity, IMD domains (excluding health), and practice and time fixed effects. Marginal effects on *PICT×Post* reported.

†Standardised mean difference.

‡Significant at p<0.05.

§Zero-inflated negative binomial model based on admission events. A&E, accident and emergency; ACSCs, ambulatory care sensitive conditions; IMD, Index of Multiple Deprivation; NBREG, negative binomial regression.

#### Robustness check

All of the estimates withstood the addition of a practice-specific time trend.

#### Stratification by risk score

We observed no relationship between risk score and time of recruitment into the intervention. Observing the plots of risk score versus total postintervention admissions, however, we see that there does appear to be a relationship between a higher risk score and increased non-elective admissions, A&E visits, total cost of secondary care services, admissions for ACSCs, inpatient length of stay and inpatient 30-day readmissions (see online supplementary material appendices). This implies that the risk score is a good predictor of these future admission types, as expected.

Results of the DDD analysis, however, indicate that those patients with a higher risk score did not benefit more from the intervention, and instead showed statistically significant increased inpatient non-elective admissions (0.0208 per patient per month; 95% CI 0.0083 to 0.0333. ES: 0.09), A&E visits (0.0363; 0.0128 to 0.0598. ES: 0.09) and inpatient length of stay (0.3071; 0.0592 to 0.5549. ES: 0.06—see online supplementary material appendices for full list of DDD estimates) compared with others. Again, the ESs indicate these increases were slight.

### Practice-level analysis

#### Sample characteristics

Table 5 shows the practice characteristics of the intervention and control practices included in the analysis (comparing the practices which joined the intervention in wave 1 with those that joined the intervention at a later date). On average, the practices are very similar, with wave 1 practices with a slightly higher proportion of older patients, and a slightly more even male/female split.

**Table 5:** Practice characteristics (wave 1 compared to later joining practices)

Mean (unless otherwise indicated)	PICT—wave 1 (SD)	Controls—later joining (SD)	SMD
N	12	18	
Male (proportion of practice)	0.52 (0.04)	0.54 (0.05)	0.04
Over 65 years (proportion of practice)	0.09 (0.03)	0.07 (0.04)	−0.15
IMD 2010	38.5 (10.7)	37.4 (7.7)	−0.12
Practice list size	6022.9 (2656.2)	6879.1 (3503.9)	0.27
GPs per thousand	0.6 (0.1)	0.8 (0.5)	0.29

GPs, general practitioners; IMD, Index of Multiple Deprivation; PICT, Practice Integrated Care Team; SMD, standardised mean difference.

Table 6 shows the crude absolute differences in mean outcome measures (per 1000 patients per month) observed between the wave 1 PICT practices and those practices joining at a later date (shown as ‘controls’ for illustration purposes), preintervention and postintervention. As for the DD results in the section below, a negative estimate indicates a relative decrease in admissions for PICT practices compared with controls. For satisfaction outcomes, a positive estimate indicates increased satisfaction for the intervention practices compared to usual care.

**Table 6:** Average absolute outcomes per 1000 patients per month by PICT (wave 1 compared to practices joining the intervention at a later date), preintervention/postintervention

Outcome	Unadjusted means (per 1000 patients per month)—wave 1 PICT compared to later joining			Unadjusted intervention effect (difference per 1000 patients per month)
	Pre (before 2012m11)	Post (after 2012m11)	Difference	
*Primary outcomes*				
Inpatient non-electives				
Controls	6.40	6.61	0.21	
PICT	8.36	8.07	−0.29	−0.50
Inpatient electives				
Controls	6.19	6.66	0.47	
PICT	8.58	8.51	−0.07	−0.54
Outpatient admissions				
Controls	87.64	97.33	9.69	
PICT	116.86	127.11	10.25	0.56
A&E visits				
Controls	25.91	28.64	2.73	
PICT	31.42	34.11	2.69	−0.04
ACSCs				
Controls	0.59	0.66	0.07	
PICT	0.85	0.85	0	−0.07
Re-admissions (30 days)				
Controls	0.87	0.84	−0.03	
PICT	1.22	1.13	−0.09	−0.06
*Secondary outcomes*				
Total cost of secondary care services (£)				
Controls	29157.28	30530.70	1373.42	
PICT	38923.43	39167.17	243.74	−1129.68
Length of stay (days)				
Controls	60.32	50.97	−9.35	
PICT	77.24	66.21	−11.03	−1.68
Patient satisfaction (general)				
Controls	0.35	0.38	0.03	
PICT	0.38	0.37	−0.01	−0.04
Patient satisfaction (LTC specific)				
Controls	0.13	0.14	0.01	
PICT	0.14	0.16	0.02	0.01

A&E, accident and emergency; ACSCs, ambulatory care sensitive conditions; LTC, long-term condition; PICT, Practice Integrated Care Team.

#### DD parallel pretrends

We identified no significant differences between pretrends for any outcome at the practice level. These data satisfy the parallel trends assumption, with no indication of bias.

#### DD results

Table 7 shows the DD analysis results at the practice level. After adjustment for practice and time fixed effects, the difference for inpatient non-elective admissions was significant, with an estimated −0.63 admissions per 1000 patients per month (95% CI −1.17 to −0.09) for PICT practices compared with control practices.

**Table 7:** Practice-level adjusted model results

Outcome	Adjusted* intervention effect (95% CI) (difference per 1000 patients per month)	Effect size†
	Linear regression model	
Primary outcomes		
Inpatient non-electives	−0.63 (−1.17 to −0.09)‡	−0.24
Inpatient electives	0.19 (−0.47 to 0.86)	0.07
Outpatient visits	−2.80 (−9.84 to 4.24)	−0.08
A&E visits	−1.32 (−3.52 to 0.89)	−0.13
ACSCs	−0.04 (−0.15 to 0.06)	−0.08
Readmissions (30 days)	−0.10 (−0.25 to 0.05)	−0.16
Secondary outcomes		
Total cost of secondary care services (£)	−505.73 (−2763.35 to 1751.89)	−0.04
Length of stay (days)	−0.24 (−7.56 to 7.08)	−0.01
Patient satisfaction (general)	−0.03 (−0.09 to 0.02)	−0.24
Patient satisfaction (LTC specific)	0.01 (−0.04 to 0.05)	0.14

N=1650 observations; 30 practices (period November 2010–March 2015).

*Adjusted for practice and time fixed effects with robust SEs.

†Standardised mean difference.

‡Significant at p<0.05.

A&E, accident and emergency; ACSCs, ambulatory care sensitive conditions; LTC, long-term condition.

The practical significance, as evidenced by the ES of −0.24, suggests a small effect of PICT on inpatient non-elective admissions at the practice level.

#### Robustness check

Following our robustness check, including a practice-specific time trend, the estimate for inpatient non-electives was no longer significant: −0.52 (−1.05 to 0.01. ES: −0.20). This may suggest that the intervention effect has absorbed some differences between treated practices due to an underlying practice-specific time trend (which can happen when policies are implemented at different points in time in different units, ie, the practice time trend which was occurring already can drive the results, so once we control for this, the estimated effect is driven towards zero).^18^ The ES, however, remained similar to the result reported above.

We were unable to predict wave 1 entry from the characteristics we included in our logistic regression model. Thus, we conclude that selection bias into early adoption, based on these characteristics at least, was minimal. However, this does not preclude the presence of selection bias based on unmeasured characteristics.

When we removed wave 1 practices (assuming these to be at most risk of selection bias, if it did indeed occur), statistical power was reduced (as expected), and the SEs of our estimates were inflated. Subsequently, we found no significant results following this robustness check. The estimate for inpatient non-elective admissions nevertheless remained negative (ie, in favour of the intervention—see online supplementary material appendices for full list of estimates following this robustness check).

## Discussion

For direct effects of the intervention, this study finds some statistically significant differences between groups, although effects are very small. The results of our DDD analysis show that even the highest risk patients (as defined by the risk prediction tool) treated did not benefit from the intervention, and in fact admissions for a number of outcomes (inpatient non-electives, A&E visits and inpatient length of stay) increased slightly for these patients.

Additional analysis at the practice level finds indications of potentially small positive spillover effects of integrated working at a higher system level. In particular, we identified a possible reduction in inpatient non-elective admissions (which, however, did not hold up to our robustness check). However, even if these effects are caused by the intervention, which this study cannot prove beyond doubt, the absolute difference observed in the analysis is small.^26^ For an average practice of approximately 6000 patients, this would equate to an estimated difference (not an absolute reduction) of −45.6 (95% CI −84.0 to −6.6) inpatient non-elective admissions in a year compared to usual care. If we estimate the average cost of an inpatient non-elective admission to be £1489,^28^ this would potentially translate to a £67 898 (95% CI £125 076 to £9827) difference compared to usual care, before accounting for intervention costs. While we did not have data on the precise intervention costs of PICT, the national Directed Enhanced Service (DES), which incentivises similar case management interventions, paid an average-sized practice £5175 for implementing the intervention in 2013/2014.^29^ This extra incentive cost of course does not account for actual additional costs of running the intervention, for example, physician time, overheads and opportunity cost of a fairly time-intensive intervention, which would also need to be considered. Additionally, our analysis found no significant effect on total secondary care costs realised during the study period, with a presumable increase in primary care costs to run the intervention (although we did not have data available on primary care costs, so cannot say for certain). Therefore, beyond the cautions we have identified for this potential spillover benefit (ie, absence of a primary effect, and not holding up to robustness checks), cost-effectiveness of the intervention remains questionable.

### Comparison of direct and spillover effects

The apparently contradictory findings at the two levels analysed merit specific discussion. First, it is worth highlighting the small proportion of patients managed by the PICT teams directly (a stipulated 2% of each practice's highest risk adult patients). The final pool of intervention patients we analysed (n=2049), therefore, only constitutes 1.04% of the patient population in the 30 practices. The likelihood of the direct effects of the intervention being a driver for practice-level results in terms of numbers treated is therefore negligible.

Second, the patients that were targeted directly by the intervention are by definition the highest risk, and potentially beyond the means of a medical intervention causing significant impact at all. This may be particularly true in the short term, for exacerbation of what are (frequently many) LTCs.^30^ Our DDD analysis adds evidence to this effect. Perhaps then, the lower risk patients in the practice would be more likely to benefit from multidisciplinary working.

Additionally, some qualitative work commissioned by the CCG separately reveals that some features of the intervention at the patient level did not occur exactly as planned. For instance, there have been problems with the implementation of the shared summary record through Graphnet, meaning the MDT case management may not have been delivered exactly as planned in every detail (beyond the practice changes introduced by the MDTs in general, and of course the case management those high-risk patients received).^31^ So, if the main driver of results was the MDT working, we may plausibly expect these effects to differ by risk group (ie, the general practice being on average at lower risk).

Finally, direct and spillover effects may plausibly act through distinct mechanisms. There are some indications of wider system effects of integrated care in the literature. For example, good team ‘climate’ (ie, professional integration)^14^ has been linked to superior clinical care for a number of LTCs,^32^ although evidence of causation is currently lacking.^33^ This is one potential mechanism that the MDT spillover effects could act through. Spillover effects, therefore, may not be dependent on the numbers captured by MDTs directly, because they go via the GP and wider care team. If practices ‘do’ MDT for a few patients, it may influence their care for everyone.

### Strengths and weaknesses of the study

Our method of analysis, DD, is a robust method under certain conditions that we tested.^11^ We only saw potential bias indicated by non-parallel preintervention trends for a single outcome measure at the individual level (outpatient visits), and we employ robustness checks beyond the primary analysis models. The method allows testing of a complex intervention in routine practice, with potential for greater external validity and generalisability of the findings.^12^

Our results at both levels are plausible. At the individual level, we observed very little differences between the groups, as we would expect from previous literature around this intervention type.^6^ At the practice level, the effect we observed was on an outcome (inpatient non-elective admissions) the intervention aimed to affect.^16^

However, our study does suffer from a number of weaknesses. Unfortunately, due to the implementation of the intervention, we were not able to access individual-level data until before the point where nearly all practices implemented the intervention. This is due to an initial problem at the CCG of consenting data use for those individual patients initially included early in the intervention. This limits our ability to ascertain whether the initially recruited patients at each practice were significantly different, or benefited more or less than those recruited later to the intervention. It also prevents direct comparison of the results we saw at the practice level with those at the individual level over exactly the same period of time and limits our ability to look at any longer term effects of the intervention at the individual level. Furthermore, if spillover effects did indeed affect other patients in the practice, then the individual-level effects may be driven towards the null. This is similarly true for the DDD analysis conducted. However, these spillover effects were not strongly indicated at the practice level.

With the intervention so widespread (particularly important for an intervention incentivised nationally), we were extremely careful to choose our comparators (a crucially important step in DD analysis). We chose practices (within the same CCG) for which we knew for definite their intervention status at any time point for the practice-level analysis. Nonetheless, practices volunteered for the intervention, which can potentially introduce some selection bias at the practice level. However, we estimate this possible selection effect to be minimal based on observable practice characteristics. A common limitation of non-experimental studies, however, is we cannot discount differences based on unobservables. Adding practice fixed effects controls for any differences between practices that persist over time, as well as any hospital-level changes during the period that affect all practices.

At the individual level, we matched patients using propensity scores within the CCG achieving the necessary parallel pretrends. However, the intervention patients are selected for their immediate risk, while the control patients were selected based on their matched risk at an earlier date, which may have subsequently subsided (and hence be the reason they were indeed not recruited to the intervention). With ‘risk’, and so recruitment, defined on time-variant indicators, and so transient over time, there is potential for some bias in favour of the control group for the individual-level results in this analysis. However, with patients well matched at the initial start date, we expect to have minimised this bias.

An important weakness, constrained by the data available to us, is we were not able to analyse outcomes beyond secondary care utilisation and total cost of secondary care. While these utilisation outcomes reflect well the explicit aims of the intervention, they do not allow for a broad representation of the intervention in terms of other important potential outcomes—for example, patient health, quality of life and satisfaction with care. These additional measures could be considered when making commissioning decisions, although they were not the primary stated aim.

### Results in relation to other studies

Our recent systematic review and meta-analysis looking at similar interventions likewise showed little effect across relevant health system outcomes for those involved in the intervention directly (ie, non-significant estimated pooled ES of 0.04 for secondary care use in the short term, and −0.02 in the long term).^6^ However, the review did show a clear benefit in terms of patient satisfaction for these patients (statistically significant estimated pooled ES of 0.26 in the short term, and 0.35 in the long term). We were unable to replicate this finding in this study, perhaps due to the data available to us that only allowed us to look at this domain at the practice level, which is likely to be less sensitive. We hypothesised from the results of our review's subgroup analyses that case management by an MDT and involving a social worker may be more effective than other examples also included in the review (eg, single nurse case manager). Results of this subsequent study do not support this previous hypothesis. However, we also suggested that ‘low-strength’ primary care systems^17^ may benefit more from the intervention (where case management may substitute for a strong primary care system). This may explain this deviation from the results of our review, which drew on evidence predominantly from a ‘low-strength’ primary care country (USA).

Looking at spillover effects from MDT case management was a strength of this paper.^14^ Only a few other studies have looked at spillover effects, most notably, evaluation of the Evercare intervention.^34^ However, Evercare used only a single case manager, where we might not expect to find large effects, and the study identified no spillover.^34^ Analysis of MDT case management in the English ‘Integrated Care Pilots’ (ICP) likewise looked at direct and spillover effects. Roland *et al* identified an increase in emergency admissions and a decrease in elective admissions and outpatient attendances at the individual level. At the practice level, they identified a slight reduction in outpatient attendances. It is, however, difficult to compare these results directly with this study, with the ICP analysis evaluating six separate sites in combination, each offering slight alterations of MDT case management to different populations. Nevertheless, key differences that stand out include the presence of a social worker in the case management team in this intervention (only two smaller sites in the ICP identified input from a social worker); physical MDT meetings in this intervention rather than ‘virtual ward’ rounds (as in the ICP sites); and the GP as clinical lead in this intervention, rather than the primarily nurse-led interventions in ICP sites.^35^

### Implications for clinicians and policymakers

This study provides further evidence of the limited effectiveness of MDT case management aimed at generally ‘at-risk’ patients as a tool to reduce care utilisation. MDT case management targeted at high-risk patients importantly does not achieve its primary aim: reducing emergency admissions for those high-risk patients directly managed. Therefore, there may be better alternatives to this intervention, which may be other forms of case management targeted at specific conditions, which have some evidence of beneficial results—for example targeting mental health.^36^ Aiming at a very small number of high-risk patients may never alleviate health system pressures alone,^30^ and even the potential spillover effects of increased professional integration that may result may not be of sufficient magnitude to achieve the desired effects.

Going beyond the case management model to a more population-based approach may therefore be another avenue to explore, for example, colocation of services, or integrated electronic health records for all patients rather than just a high-risk cohort—interventions further removed from the service delivery level, but which may be regarded as a key foundation for multidisciplinary professional communication and working. We have shown here that this greater professional integration may have scope for improving measurable health system outcomes.

### Future research

More work is needed to confirm these initial findings of potentially beneficial spillover effects, particularly qualitative work and process evaluation identifying plausible mechanisms. These did not stand up to our robustness checks in this analysis; however, the indication was always in the direction of favouring the intervention practices with regard to decreasing non-elective admissions at the practice level. Where it is possible, future studies looking at models of integrated care should consider spillover effects.

If commissioning bodies consider evaluation using similar robust, but cost-effective methods in the future, they should be planned from the beginning, where potential bias (discussed above) could be easily avoided. For example, a randomised stepped-wedge design may be an appropriate alternative.^37^

While we improved on previous literature by including a measure of multimorbidity in our study, we only included the most basic of these, a simple count of diseases.^38^ Our future research will explore outcomes stratified by different ‘types’ of multimorbidities, to observe if the intervention can be better targeted for the patients it directly affects, providing a more effective and efficient method of exploiting the potential for wider system effects.

## Conclusions

We show that MDT case management does not fulfil its primary aim, preventing emergency admissions for the high-risk patients it targets. This accords with our previous findings. We show here that the highest risk patients (as identified by the risk tool) receiving the intervention in fact slightly increased admissions in many domains targeted for decrease by the intervention. We do, however, show some indications of beneficial spillover effects of MDT working at the practice level worthy of further exploration. The results highlight the importance of ongoing work on effective ways of avoiding admissions.^36^
