# Supplementary material for: Effectiveness of multidisciplinary team case management: difference-in-differences analysis
Source: BMJ Open. 2016 Apr 15;6(4):e010468. doi: 10.1136/bmjopen-2015-010468 (PMC4838740; doi:10.1136/bmjopen-2015-010468)
Supplement: Supplementary data [file bmjopen-2015-010468supp.pdf]

Appendices

APPENDICES 1

INDIVIDUAL-LEVEL METHODS EXTENSION 1
PRACTICE-LEVEL METHODS EXTENSION 5
MULTIMORBIDITY MEASURE 7
MISSING DATA 9
AMBULATORY CARE SENSITIVE CONDITIONS (ACSCS) 10
PATIENT SATISFACTION 11
PARALLEL TRENDS GRAPHS 12
DIRECTED ACYCLIC GRAPHS (DAGS) 21
PRACTICE-LEVEL ROBUSTNESS CHECK ESTIMATES (AFTER REMOVAL OF WAVE 1 PRACTICES) 24
RISK SCORE STRATIFICATION RESULTS (INDIVIDUAL-LEVEL) 25
RISK SCORE DDD ESTIMATES 30
REFERENCES 31

Individual-level methods extension

The parallel trends assumption was not fulfilled when we compared the PICT patients to all possible control patients in the CCG. We therefore had to match intervention patients with potential controls from within the CCG using one-to-one propensity score. We matched using characteristics for which the intervention patients were selected in implementation (based on age; sex; index of multiple deprivation (IMD) 2010; total multimorbidity count previous to the first available start date of our intervention patients; previous inpatient, outpatient and A&E attendance in the previous year before the first intervention patient start date).[1]

As recommended,[2] prior to matching, we first imputed missing data for IMD based on the other complete variables, using multivariate normal multiple imputation (we used the STATA command 'mi impute' and used the average of

10 imputations).[3 2] We used STATA's 'pscore' and 'psmatch2' commands for propensity score matching.[4 5]

When we identified any control patients who had died before their matched start date, we eliminated these controls from the matching dataset (as well as all those intervention and control patients already matched adequately) and returned to this reduced matching pool to repeat the propensity match for the remaining intervention patients. This process was repeated twice, until all intervention patients were adequately matched to a living patient at their start date (round 1, n=1982 intervention patients correctly matched; round 2, n=62; round 3, n=5; total, n=2049).

The fact that nearly all practices were already implementing the intervention prior to individual-level analysis means the risk score of the control patients is likely to be slightly lower than the risk score of the intervention patients (i.e. we would expect that the highest risk patients in each practice would have already been signed up to the intervention). However, the DD analysis technique we use does not require control and intervention groups to be exactly the same, only that they are comparable based on parallel pre-trends of each of the outcomes assessed.

Outcome measures were summed to a count per patient per month over the period September 2010 to March 2015 inclusive, to allow a 3-year pre-trend period. All summed co-variates were once again sourced from the master

dataset, together with the cumulative multimorbidity count (described in the Appendices). Missing observations were filled as detailed in the Appendices.

Analysis models were chosen based on the results of the stata 'countfit' command, which compares count models based on the Akaike information criterion (AIC), the Bayesian information criterion (BIC), and Vuong's closeness test. For the majority of outcomes, the best fitting model was the negative binomial model. The exception to this was for *total cost of secondary care services*, where we used a zero-inflated negative binomial model (inflating based on admission events). In each case, all three comparison tests agreed to the count model chosen. The equation used was:

$$y_{ijt} = \beta_1 PICT_{ijt} + \beta_2 PICT_{ijt} \times Post_{ijt} + \delta_t + \boldsymbol{xk}_{it} + \alpha_j + \varepsilon_{ijt}$$

Where:

y_{ijt} = outcome of person i in practice j in time t

$PICT_{ijt}$= dummy for treatment status

$PICTxPost_{ijt}$ = a dummy variable which equals one for treatment units in the post-treatment period, and is otherwise zero

δ_t= time fixed-effects

$\boldsymbol{xk}_{it}$ = *individual covariates* (age, cumulative multimorbidity count, IMD 2010 domains (excluding health domain))

α_j = constant with absorbed practice fixed-effects

ε_{ijt} = random error

Stratification by risk score

We were interested to observe whether those treated patients with a higher risk score, were indeed those at highest risk of future emergency admissions, and to test whether those at higher risk benefited more from the intervention.

While we were only able to access the actual risk scores of the intervention patients and not the controls, due to information governance rules, we assessed the correlation of the actual risk score of the intervention patients with the propensity score we used to match the intervention patients to controls. We found these to be moderately,[6] statistically significantly correlated ($r=0.38$; $p<0.0001$), and therefore assigned the same actual risk score of the intervention patients to their one-to-one propensity matched control for this sub-analysis.

We first looked descriptively at the association of risk score and post-intervention outcomes by calculating a single 'post-intervention' admissions value for each of the secondary care utilisation measures, and plotted this graphically against the risk score. To ensure the highest risk patients were not also those recruited first to the intervention (thus artificially inflating our simple measure for this sub-analysis), we additionally plotted each intervention patient start date together with the risk score to determine any time-varying relationship that might exist.

We assigned those intervention patients and matched controls with a risk score more than the 75th percentile to the high-risk group, and conducted the DDD

analysis. The extended equation for this extra interaction-term becomes, where the average partial effect of results for β_7 is reported:

$$y_{ijt} = \beta_1 PICT_{ijt} + \beta_2 Post_{ijt} + \beta_3 RISK_i + \beta_4 PICT_{ijt} \times Post_{ijt} + \beta_5 PICT_{ijt} \times RISK_i + \beta_6 Post_{ijt} \times RISK_i + \beta_7 PICT_{ijt} \times Post_{ijt} \times RISK_i + \delta_t + \boldsymbol{xk}_{it} + \alpha_j + \varepsilon_{ijt}$$

Where:

y_{ijt} = outcome of person i in practice j in time t

$PICT_{ijt}$= dummy for treatment status

$RISK_i$ = dummy for high risk score according to risk tool

$Post_{ijt}$ = dummy for the post-treatment period

δ_t= time fixed-effects

$\boldsymbol{xk}_{it}$ = *individual covariates* (age, cumulative multimorbidity count, IMD 2010 domains (excluding health domain))

α_j = constant with absorbed practice fixed-effects

ε_{ijt} = random error

Practice-level methods extension

The practice integrated care teams were initially introduced as an option within a mandatory (contracted and paid) Quality Process scheme in the CCG, therefore practices initially volunteered for PICT. Due to the staggered start dates of practices signing up to the PICT intervention (see **Error! Reference source not found.** in main paper), a natural experiment emerged at the practice-level. We exploited this rollout with data collapsed to the practice-level for 30 practices in

the CCG, for which we had detailed start date information. Outcomes were summed to a count per 1000 patients per month for each of the practices, and analysed over the period September 2010 to March 2015 inclusive, to overlap with the individual-level analysis.

The practice fixed-effects in our analysis model act to remove any observed and unobserved, time-invariant confounders at the practice level (e.g. should PICT practices be relatively more focussed on reducing secondary care attendance than non-PICT practices), while the time fixed-effects act to remove any fluctuations in outcome due to the general trend over time. We report the results of β_1 in the main text, together with the corresponding effect size. The equation used was:

$$y_{jt} = \beta_1 PICT_{jt} \times Post_{jt} + \delta_t + \alpha_j + \varepsilon_{jt}$$

Where:

y_{jt} = outcome of practice j in time t

$PICT_{jt}xPost_{jt}$ = a dummy variable which equals one for treatment units in the post-treatment period, and is otherwise zero

δ_t = time fixed effects

α_j = constant with absorbed practice fixed-effects

ε_{jt} = random error

At the practice-level only, due to the voluntary roll-out of the intervention, we attempted to assess the effects of selection bias using a logistic regression model. We attempted to predict the wave of entry to the intervention using the practice

characteristics recorded (including: % males; % over 65; list size; number of GPs per thousand patients; total IMD score 2010; and total % QOF achievement score).[7] As a further robustness check, we additionally re-ran the practice-level analysis excluding those practices recruited to the intervention in wave 1, assuming these to be the practices at most risk of selection bias if it did indeed occur.[8]

Multimorbidity measure

For the individual-level analysis, a multimorbidity measure was prepared from the previously recorded (period June 2006 to March 2015) inpatient admissions, for 20 chronic conditions recorded in the Quality and Outcomes Framework (QOF) (see below for list of conditions and icd-10 codes).[9-11] This measure was recorded cumulatively by month over the dataset, with a binary indicator for each condition 'switched on' by the recording in the inpatient record, subsequently staying 'on', with addition of any further diagnoses recorded at a later date. The cumulative monthly total was used in the analysis.

Although we would have preferred to use chronic conditions data recorded in primary care for our multimorbidity measure, this data was unavailable for this study. However, as high-risk patients (both the intervention and propensity matched controls), these are probably the most likely to encounter inpatient admissions (and indeed were selected for 'high risk' of admissions), so should in theory have the most complete recordings at this service level in comparison to the general population. From previous literature though, we can expect our multimorbidity measure to be less sensitive (i.e. predictably lower count)

because it comes purely from hospitalisation data.[12] Nevertheless, our multimorbidity measure gained strength from its cumulative nature, accounting for changes over the life-course. Furthermore, we calculated the count based on a list of 20 chronic conditions, deemed particularly important in the UK's NHS setting, which follows guidance from the multimorbidity literature.[13]

1. Asthma
J45-J47

2. Atrial fibrillation
I48

3. Cancer
C00–C14, C15–C26, C30–C39,
C40–C41, C43–C44, C45–C49,
C50, C51–C58, C60–C63,
C64–C68, C69–C72, C73–C75,
C81–C96, C76–C80, C97,
D00–D09, D37–D48 (Koller et al 2014)

4. Chronic kidney disease
N18

5. Coronary heart disease
I20-I25

6. COPD
J40-J44

7. Dementia
F00-F03

8. Depression
F32-F33

9. Diabetes mellitus
E10-E14

10. Epilepsy
G40-G41

11. Heart failure
I50

12. Hypertension
I10-I15

13. Hypothyroidism
E00-E03

14. Learning disability
F80-F89

15. Mental health (schizophrenia, bipolar affective disorder and other psychoses)
F20-F29, F31, F34-F39

16. Obesity
E66

17. Osteoporosis
M80-M82

18. Atherosclerosis/Peripheral arterial oclusive disease (PAOD)
I65–I66, I67.2, I70, I73.9

19. Rheumatoid arthritis
M05-M06

20. Cerebral ischemia/chronic stroke
I60–I64, I69, G45

Missing data

Any missing Lower Layer Super Output Area (LSOA) codes (n= 295) were updated with the dominant LSOA where patients live, according to their GP practice code.[14] IMD domains (excluding the health domain) were matched to the LSOA codes.

Missing panel data (where the patient had no secondary care service use in a given month) was filled with zero observations using the STATA command 'tsfill' to make a balanced panel dataset.[15] Any filled observations following inpatient

mortality were deleted for that given patient, as well as any observations giving a negative age (for the youngest patients included in the intervention/control).

All other data used was complete in the dataset.

Ambulatory Care Sensitive Conditions (ACSCs)

Ambulatory Care Sensitive Conditions (ACSCs) are specified conditions, which should be managed adequately at the primary care level and prevented from worsening to the extent that secondary care is necessary. They are deemed an indication of access to and effectiveness of primary care in a health system.[16 17] When these set conditions are not managed effectively (particularly in a national health system which should provide universal access, as is the case in the UK), a harm is befalling the patient and a safety incident can be said to have occurred.[18]

We used the NHS's definition of an ACSC.[19] We included the following icd-10 codes, when they were coded as the primary diagnosis, for an emergency admission only.

Vaccine preventable
B18.0 Chronic viral hepatitis B with delta-agent
B18.1 Chronic viral hepatitis B without delta-agent
Asthma
J45 Asthma
J46X Status asthmaticus
Congestive heart failure
I11.0 Hypertensive heart disease with (congestive) heart failure
I50 Heart failure
J81X Pulmonary oedema
I13.0 Hypertensive heart and renal disease with (congestive) heart failure
Diabetes

E10 Insulin-dependent diabetes mellitus
E11 Non-insulin-dependent diabetes mellitus
E12 Malnutrition-related diabetes mellitus
E13 Other specified diabetes mellitus
E14 Unspecified diabetes mellitus
Chronic obstructive pulmonary disease
J20 Acute bronchitis
J41 Simple and mucopurulent chronic bronchitis
J42X Unspecified chronic bronchitis
J43 Emphysema
J44 Other chronic obstructive pulmonary disease
J47X Bronchiectasis
Angina
I20 Angina pectoris
I25 Chronic ischaemic heart disease
Iron deficiency anaemia
D50.1 Sideropenic dysphagia
D50.8 Other iron deficiency anaemias
D50.9 Iron deficiency anaemia, unspecified
D51 Vitamin B12 deficiency anaemia
D52 Folate deficiency anaemia
Hypertension
I10X Essential (primary) hypertension
I11.9 Hypertensive heart disease without (congestive) heart failure
Convulsions and epilepsy
G40 Epilepsy
G41 Status epilepticus
Dementia
F00 Dementia in alzheimers
F01 Vascular dementia
F02 Dementia in other diseases
F03 Unspecified dementia
Atrial fibrillation and flutter
I48X Atrial fibrillation and flutter

Patient satisfaction

Patient satisfaction was assessed at the practice-level using data from the GP Patient Survey (GPPS). The GPPS was provided by Ipsos MORI at the individual-level.[20] The satisfaction measure was analysed using two questions from the GPPS:

1. Overall, how would you describe your experience of your GP surgery? (general satisfaction)

2. In the last 6 months, have you had enough support from local services or organisations to help you to manage your long-term health condition(s) (LTC)? (LTC-specific satisfaction)

Responses were coded to a binary variable (1 for 'Very good'/'Yes, definitely', respectively; 0 for any less positive response, with missing responses coded as missing) for each patient within each of the 30 practices. This data was collapsed (summed) to the practice level (with weights used to collapse to representative samples of practice populations), along with a variable coded as '1' for each observation, which was consequently used to weight the outcomes by number of responses received for each practice, so that no practice was over-represented in the final analysis. The 'pre-period' was three survey waves, comprising the period from July 2011 to September 2012; and the 'post-period' for all practices was two survey waves, comprising the period from January 2013 to September 2013.

Parallel Trends graphs

Pre-trends were additionally tested statistically using a continuous time linear time trend interacted with the treatment dummy, as well as multiple time dummies interacted with treatment dummy and using an f-test to assess overall significance, both methods using the pre-intervention data only. The results of these tests are reported in the main paper.

Practice-level average trends (wave 1 practices versus later joining practices)

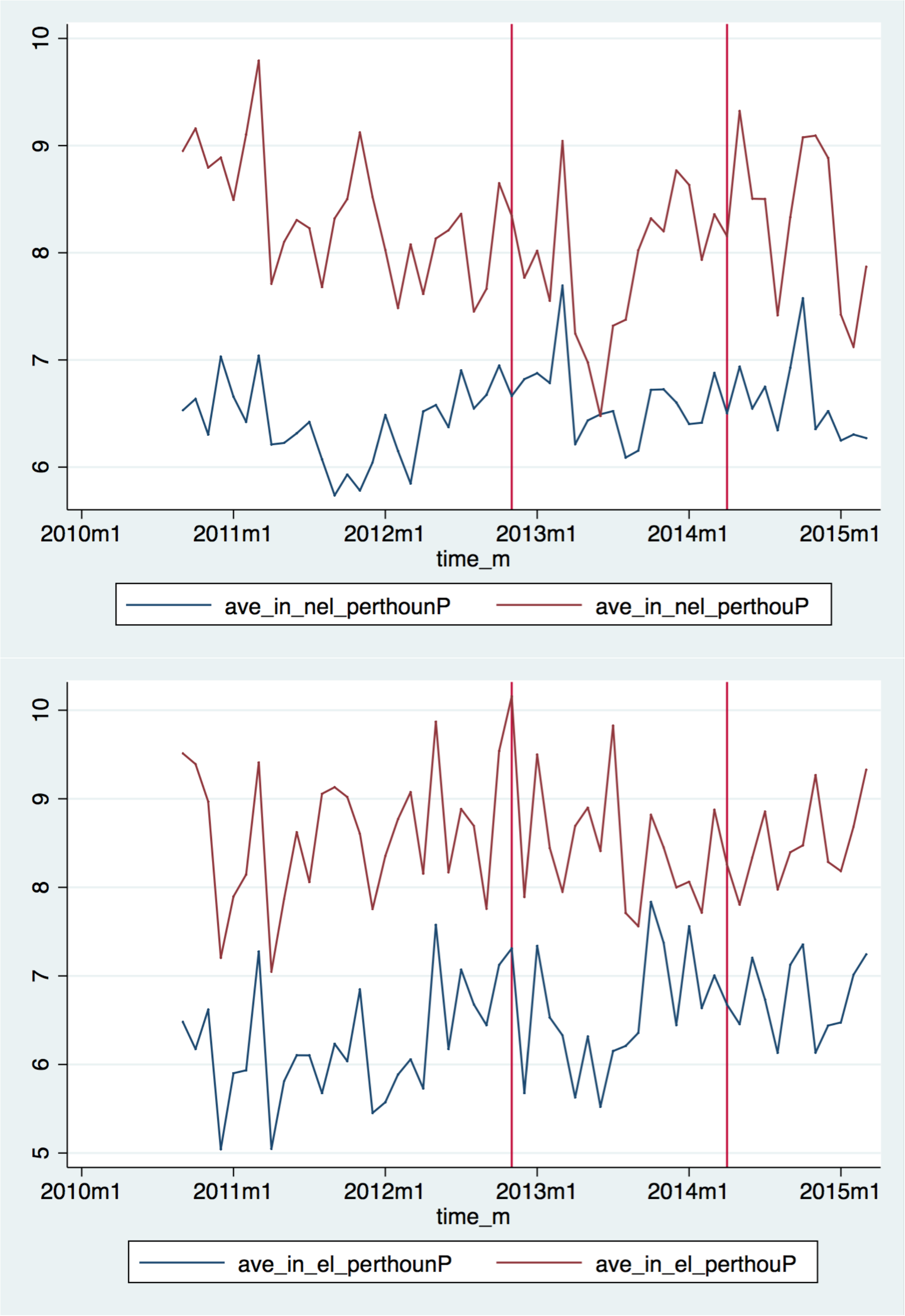

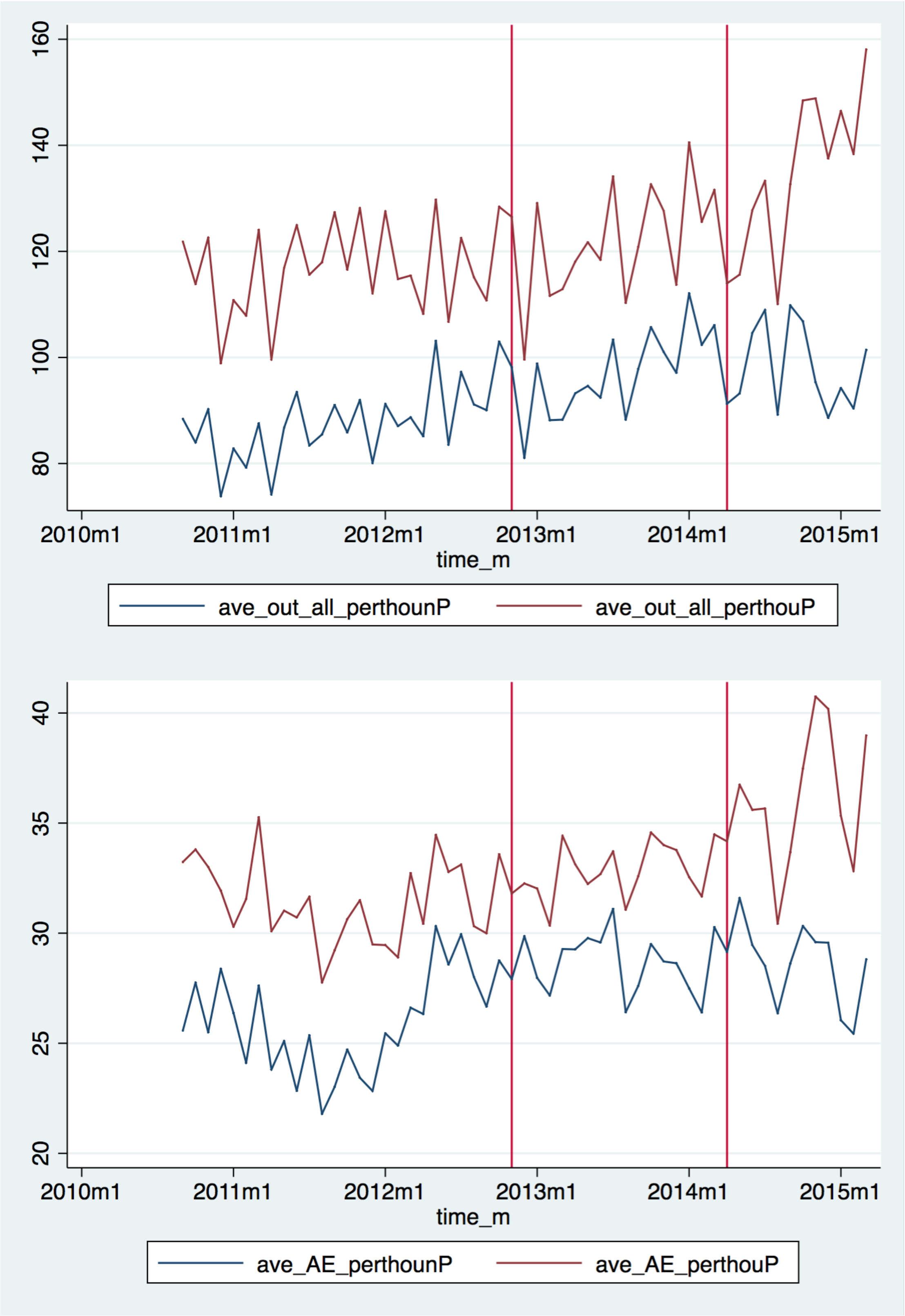

160
140
120
100
80
2010m1
2011m1
2012m1
2013m1
2014m1
2015m1
time_m
ave_out_all_perthounP
ave_out_all_perthouP
40
35
30
25
20
2010m1
2011m1
2012m1
2013m1
2014m1
2015m1
time_m
ave_AE_perthounP
ave_AE_perthouP

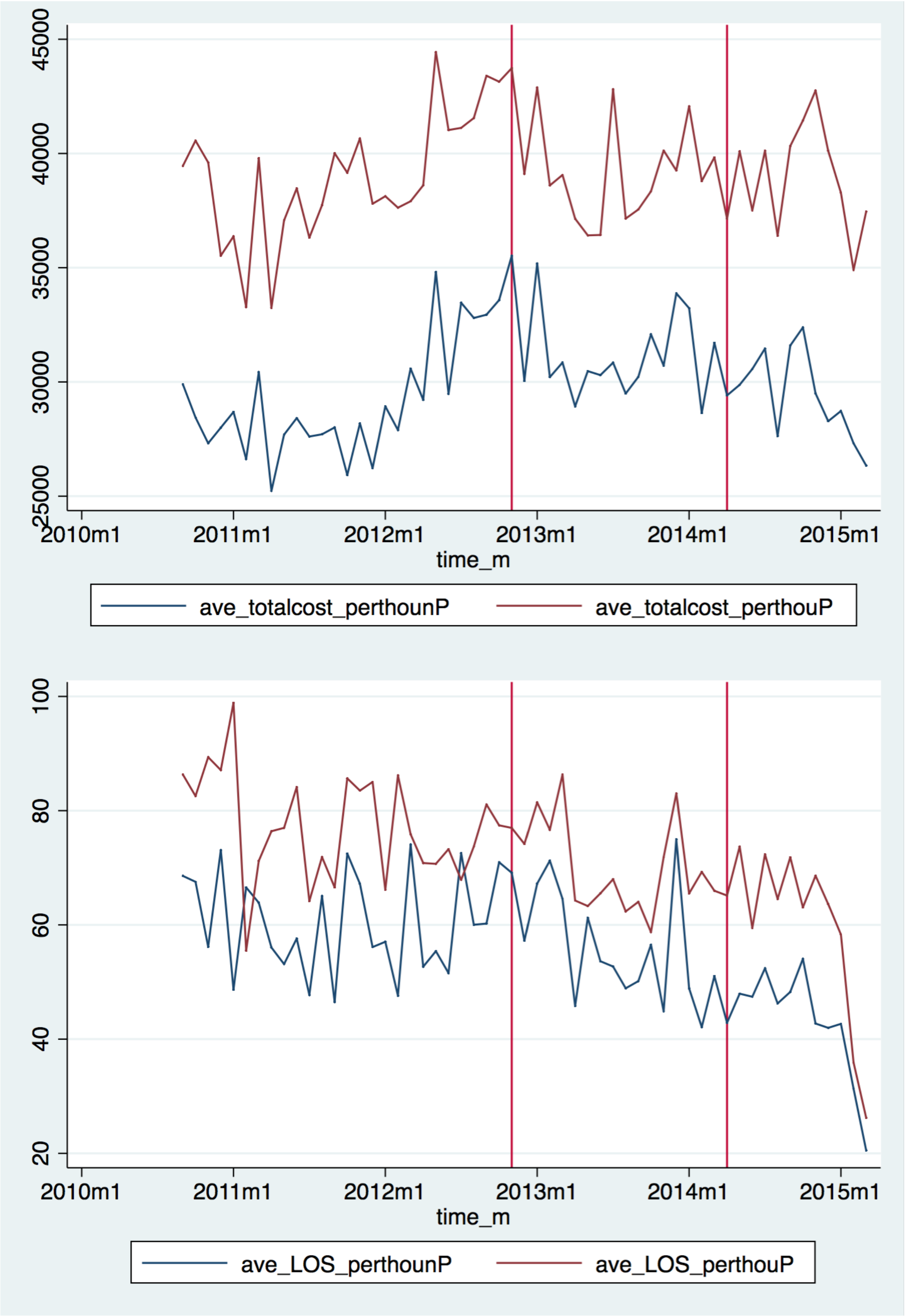
45000
40000
35000
30000
25000
2010m1
2011m1
2012m1
2013m1
2014m1
2015m1
time_m
ave_totalcost_perthounP
ave_totalcost_perthouP
100
80
60
40
20
2010m1
2011m1
2012m1
2013m1
2014m1
2015m1
time_m
ave_LOS_perthounP
ave_LOS_perthouP

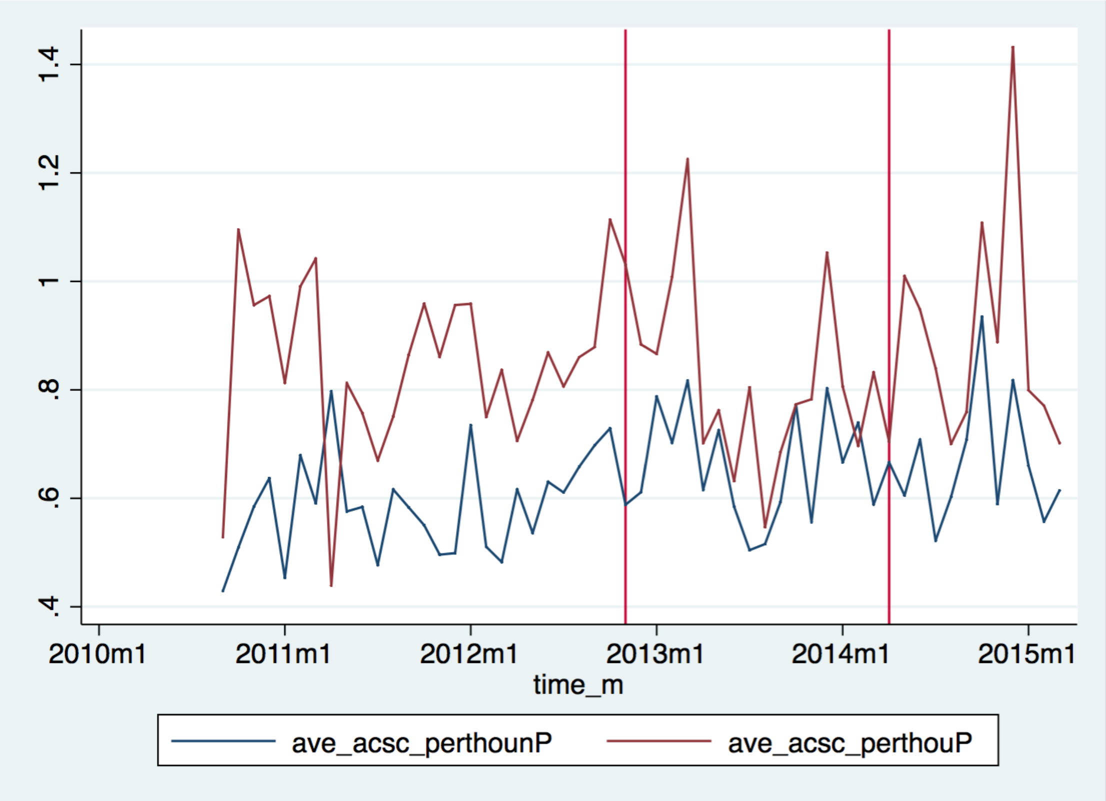
1.4
1.2
1
.8
.6
.4
2010m1
2011m1
2012m1
2013m1
2014m1
2015m1
time_m
ave_acsc_perthounP
ave_acsc_perthouP

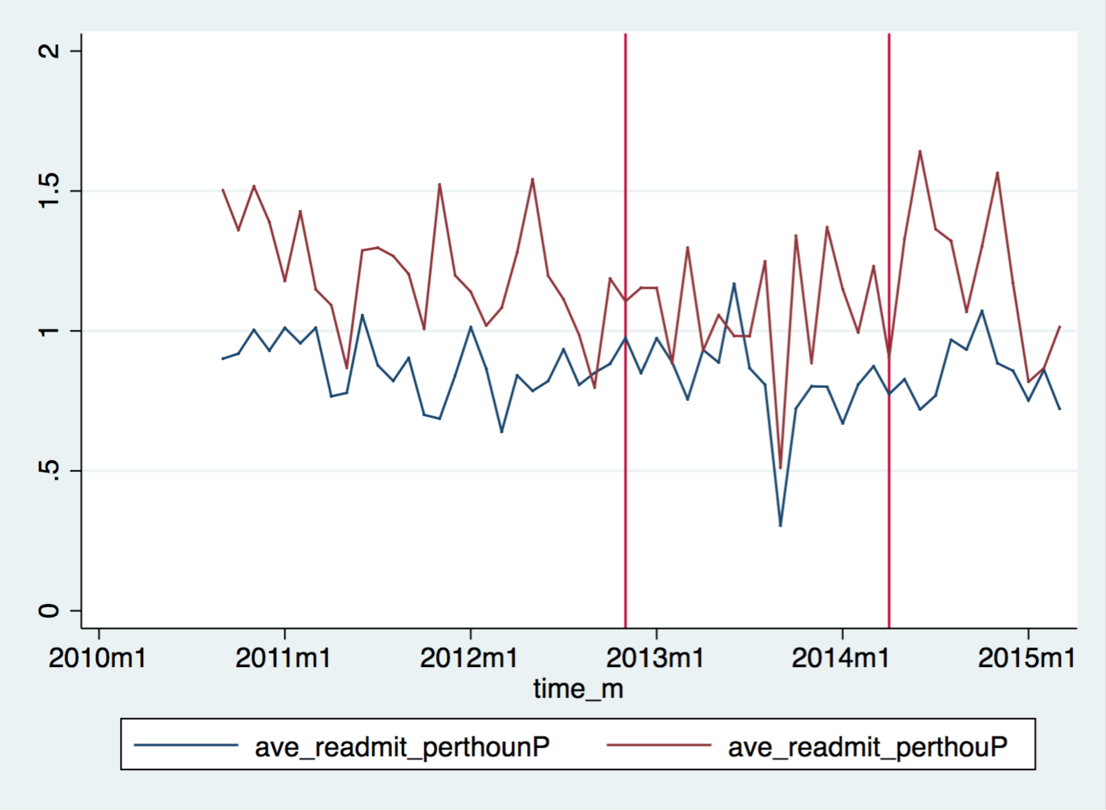
2
1.5
1
.5
0
2010m1
2011m1
2012m1
2013m1
2014m1
2015m1
time_m
ave_readmit_perthounP
ave_readmit_perthouP

Individual-level average trends

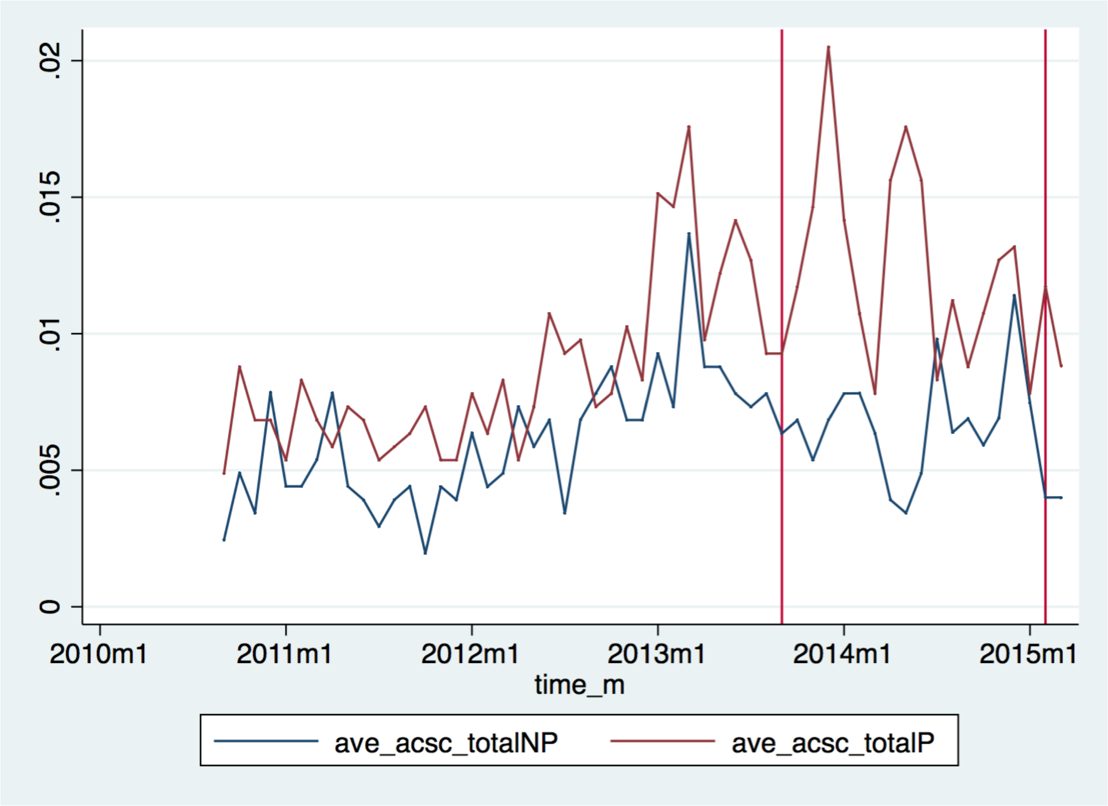

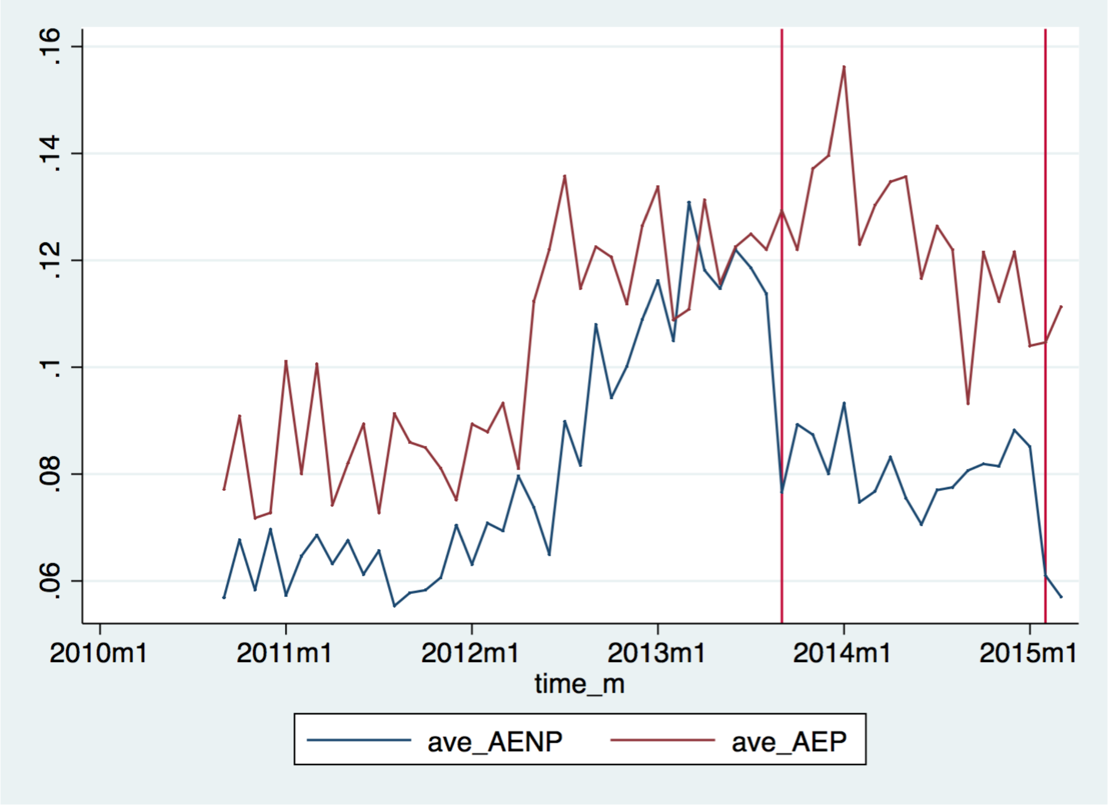

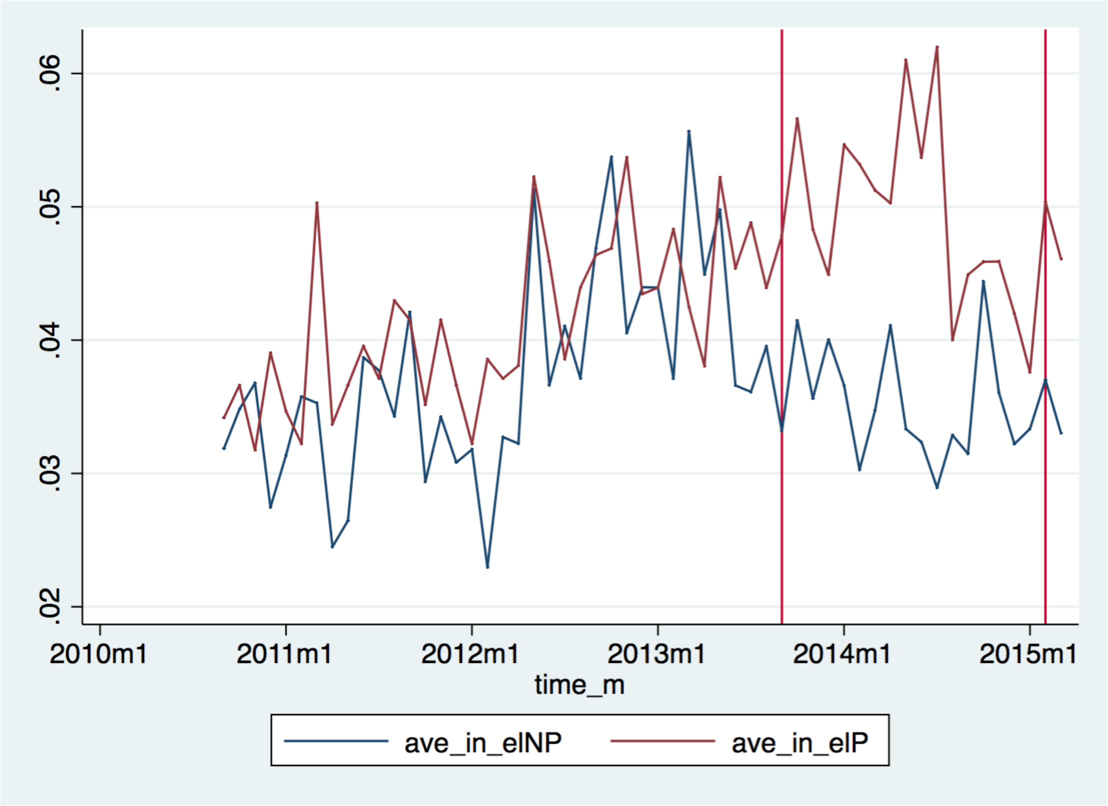
.02
.03
.04
.05
.06
2010m1
2011m1
2012m1
2013m1
2014m1
2015m1
time_m
ave_in_elNP
ave_in_elP

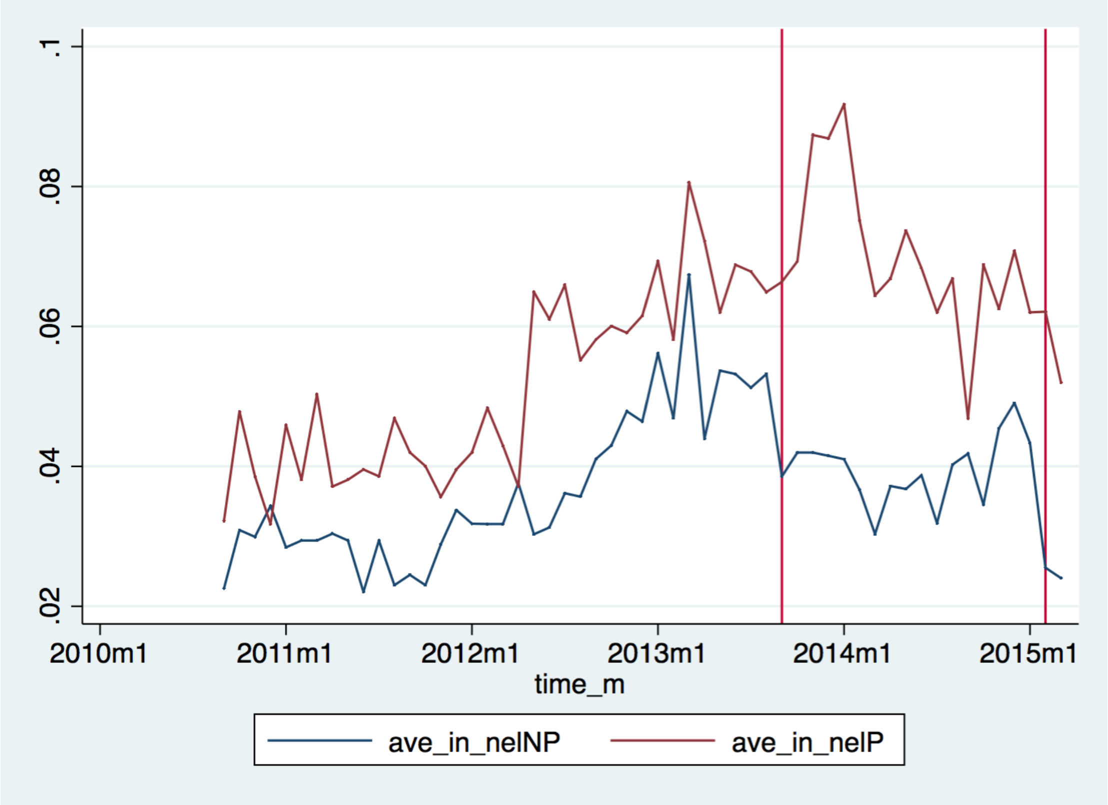
.02
.04
.06
.08
.1
2010m1
2011m1
2012m1
2013m1
2014m1
2015m1
time_m
ave_in_nelNP
ave_in_nelP

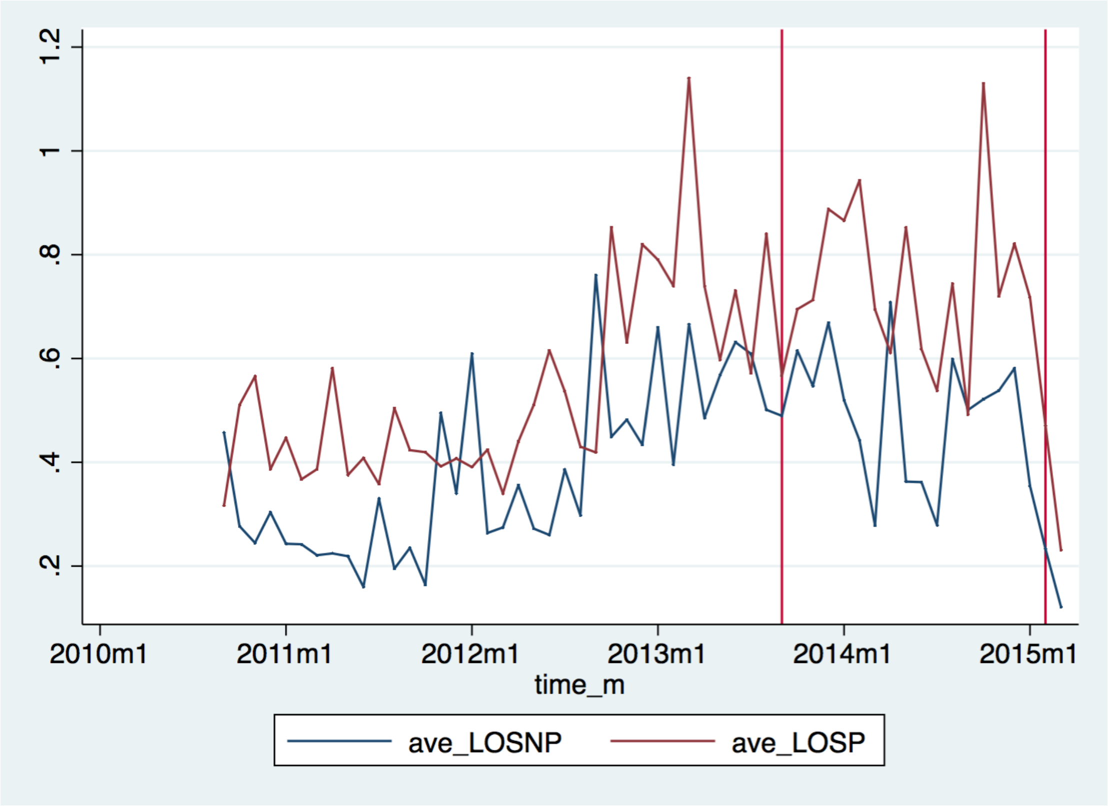

1.2
1
.8
.6
.4
.2
2010m1
2011m1
2012m1
2013m1
2014m1
2015m1
time_m
ave_LOSNP
ave_LOSP

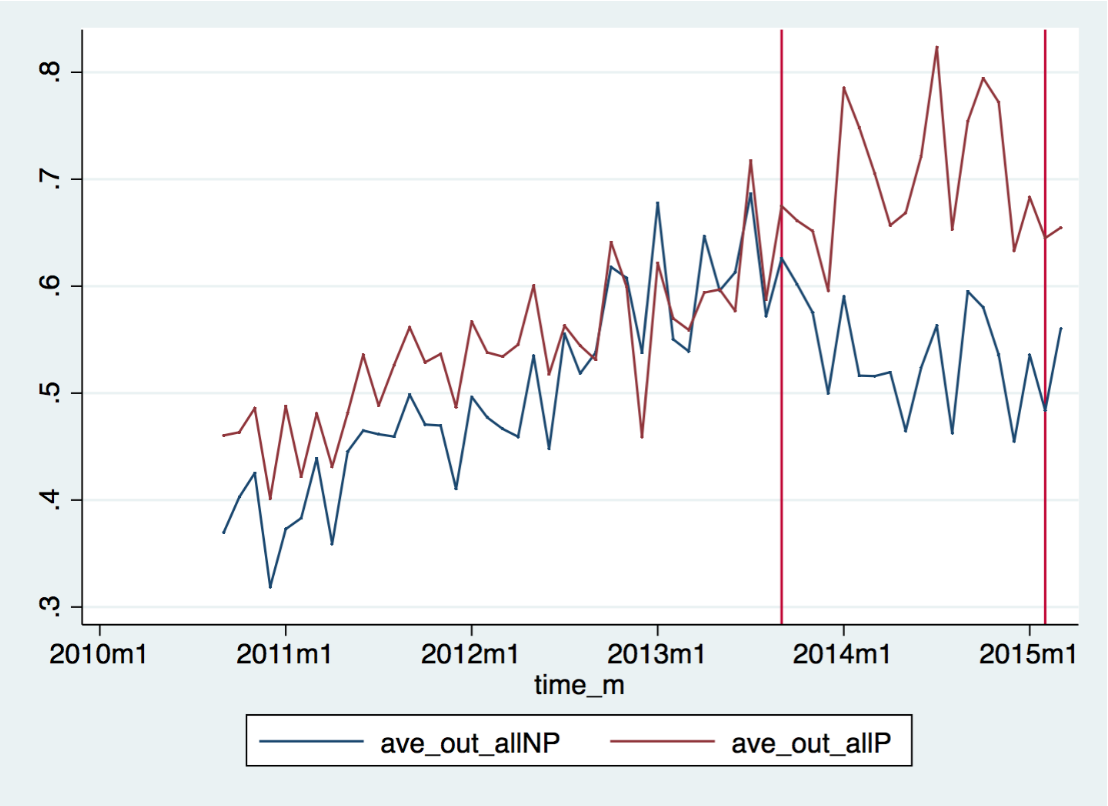

.8
.7
.6
.5
.4
.3
2010m1
2011m1
2012m1
2013m1
2014m1
2015m1
time_m
ave_out_allNP
ave_out_allP

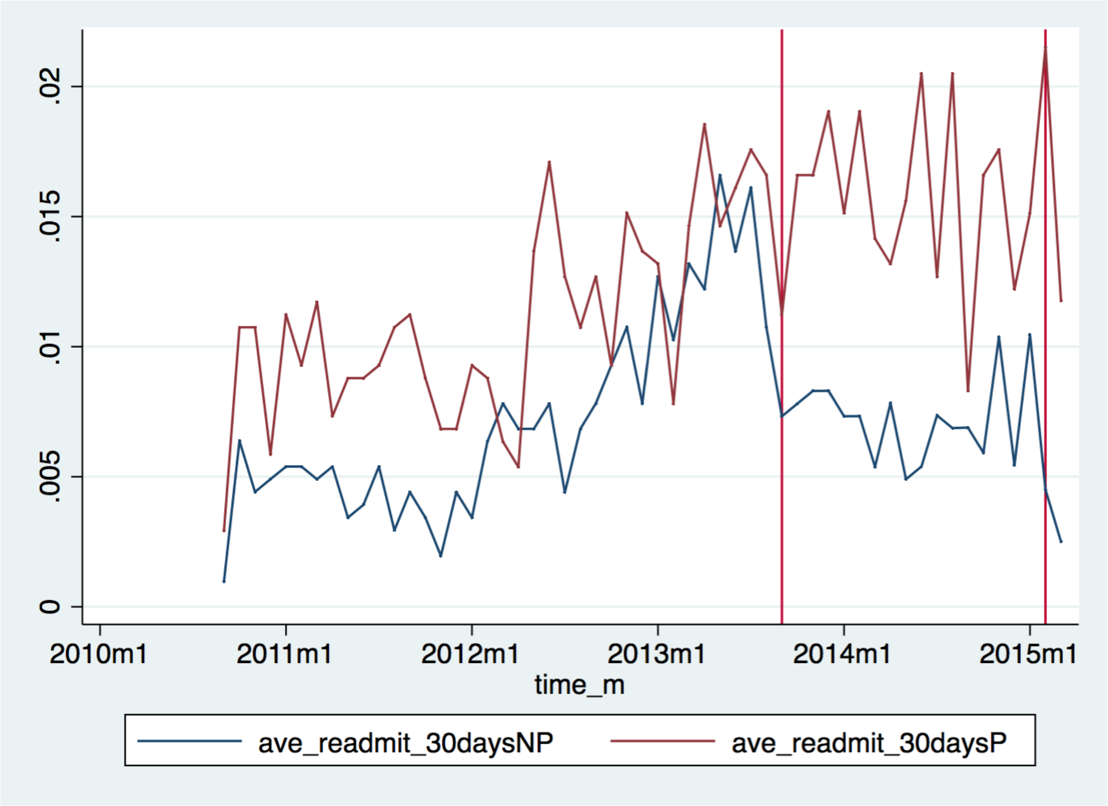

0
.005
.01
.015
.02
2010m1
2011m1
2012m1
2013m1
2014m1
2015m1
time_m
ave_readmit_30daysNP
ave_readmit_30daysP

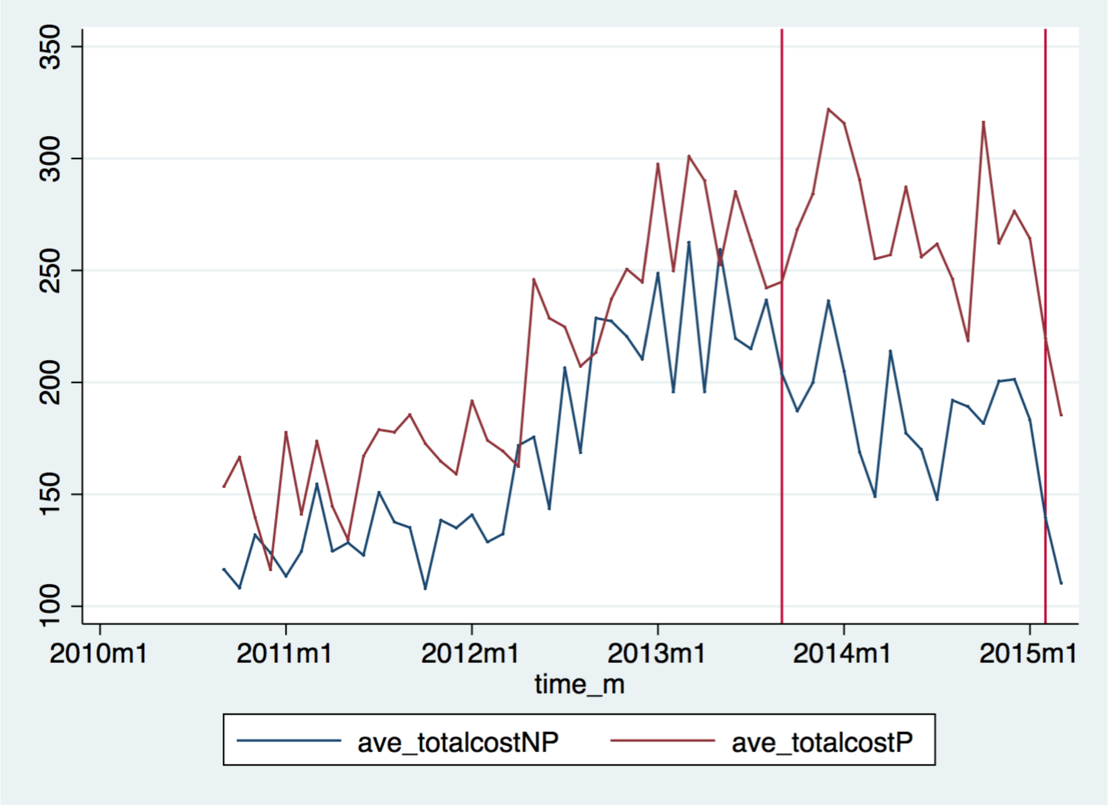

100
150
200
250
300
350
2010m1
2011m1
2012m1
2013m1
2014m1
2015m1
time_m
ave_totalcostNP
ave_totalcostP

Directed Acyclic Graphs (DAGs)

Co-variates

We adjusted for co-variates in each model, chosen based on causal diagrams, known as directed acyclic graphs (DAGs – see Appendices).[21] DAGs are graphical descriptions that require us to set down clearly our assumptions about causal relationships, and can be used to select the minimal sufficient adjustment for determining the causal relationship of interest (in this case the effect of the PICT intervention on each of the outcome measures listed above), while ensuring minimum bias.[21]

The DAGs were created using free open-access software available at http://www.dagitty.net/dags.html#.

Practice-level

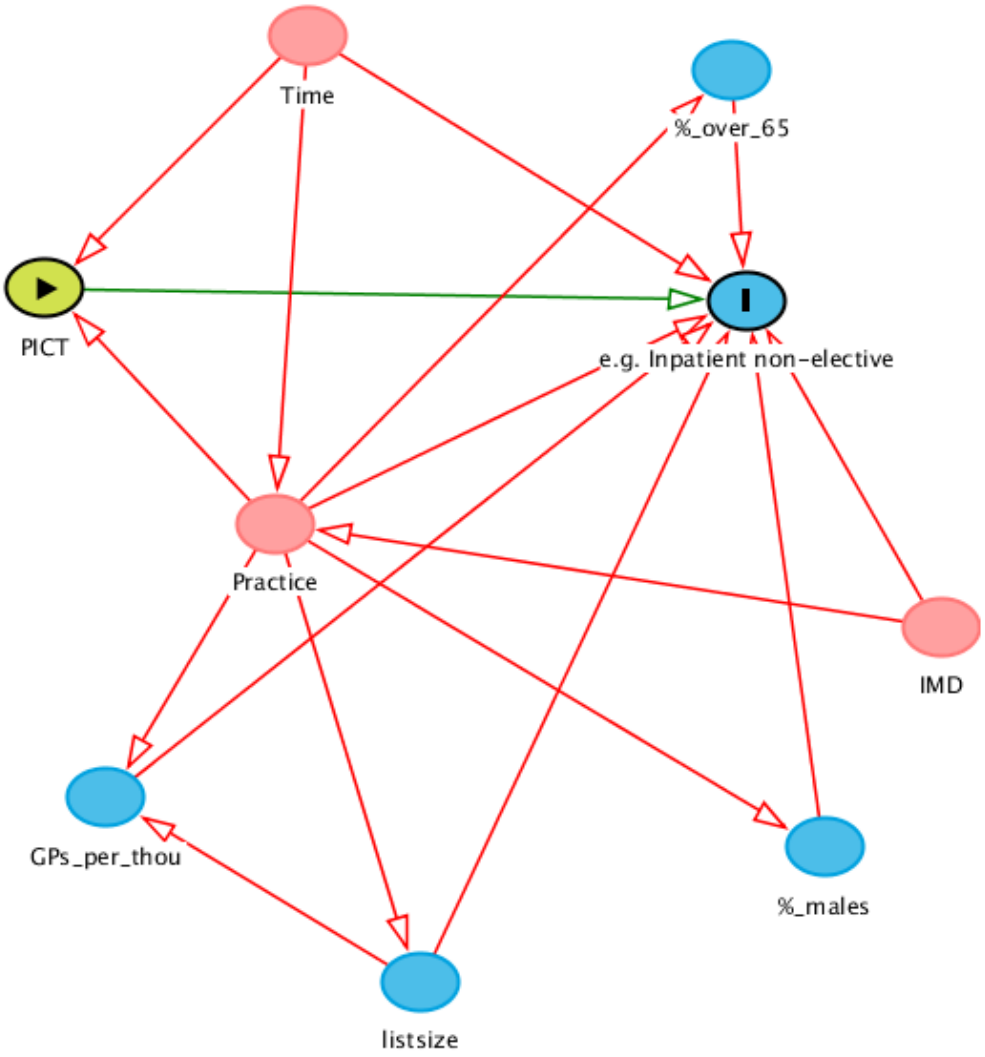

Minimal sufficient adjustment sets for estimating the total effect of PICT on e.g. Inpatient non-elective:

- Practice, Time

Individual-level

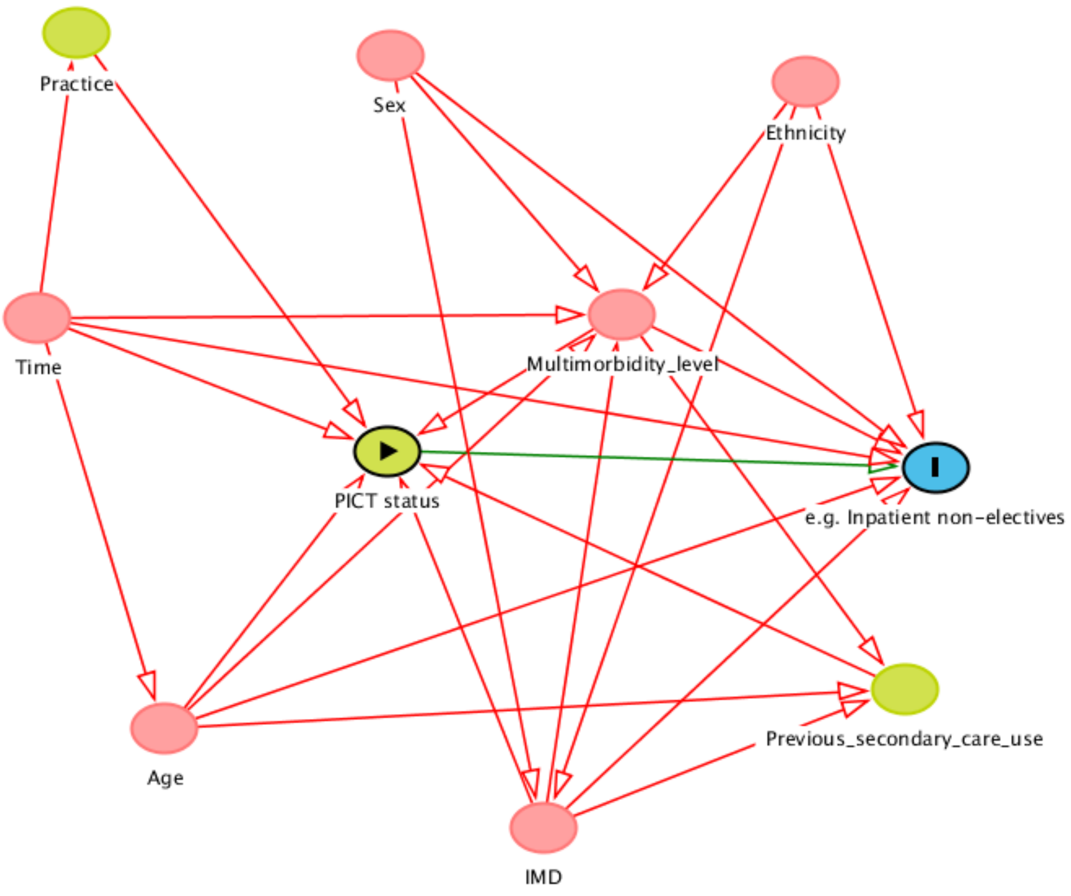

Minimal sufficient adjustment sets for estimating the total effect of PICT status on e.g. Inpatient non-electives:

- Age, IMD, Multimorbidity_level, Time

Practice-level robustness check estimates (after removal of wave 1 practices)

Outcome	Adjusted+ Intervention effect (95% CI)	Effect size*
	**Linear regression model**	
**Primary outcomes:**		
Inpatient non-electives	-0.23 (-1.02 to 0.56)	-0.09
Inpatient electives	0.04 (-0.56 to 0.64)	0.01
Outpatient admissions	0.99 (-6.17 to 8.15)	0.03
A&E visits	0.35 (-2.35 to 3.06)	0.03
ACSCs	-0.05 (-0.20 to 0.10)	-0.10
Re-admissions (30 days)	0.01 (-0.22 to 0.22)	0.02
**Secondary outcomes:**		
Total cost of 2° care services	610.36 (-2434.52 to 3655.25)	0.05
Length of stay (days)	4.03 (-4.95 to 13.02)	0.10
Patient satisfaction (general)	-0.03 (-0.10 to 0.05)	-0.24
Patient satisfaction (LTC-specific)	0.01 (-0.05 to 0.07)	0.14

+: adjusted for practice and time fixed-effects with robust standard errors
*: standardised mean difference
#: significant at p<0.05
n= 990 observations; 18 practices (period November 2010 to March 2015)

Risk score stratification results (individual-level)

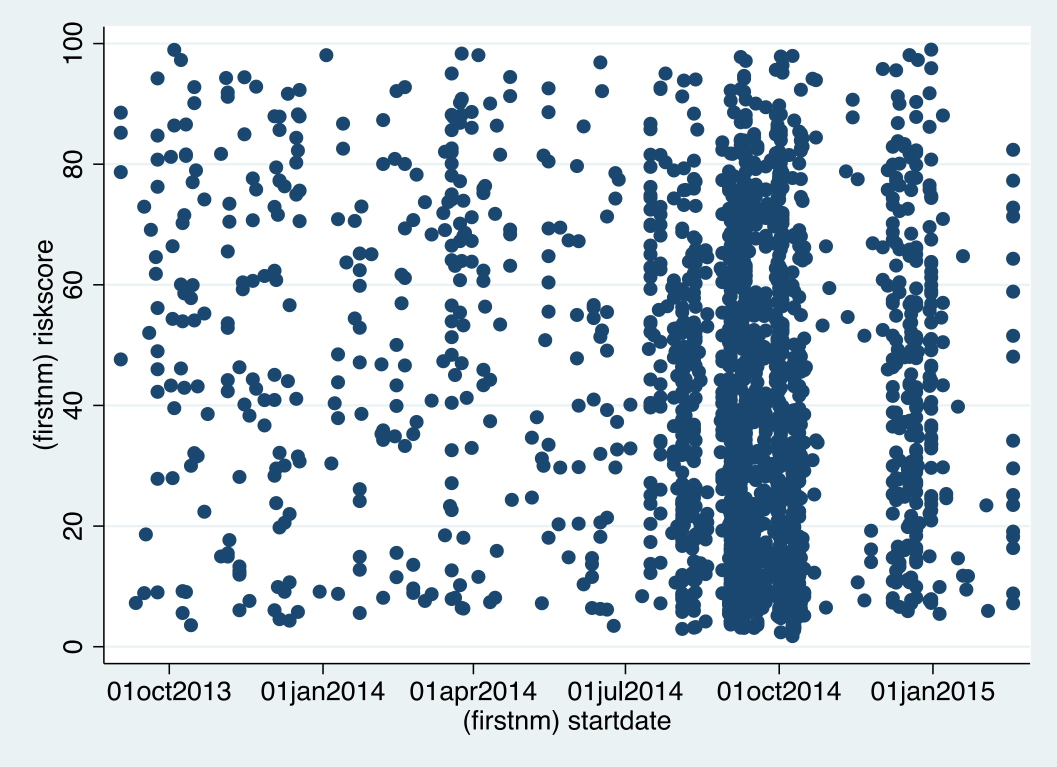

No indication of relationship between risk score and time recruited to intervention.

How well does actual risk score predict number of POST-intervention admissions?

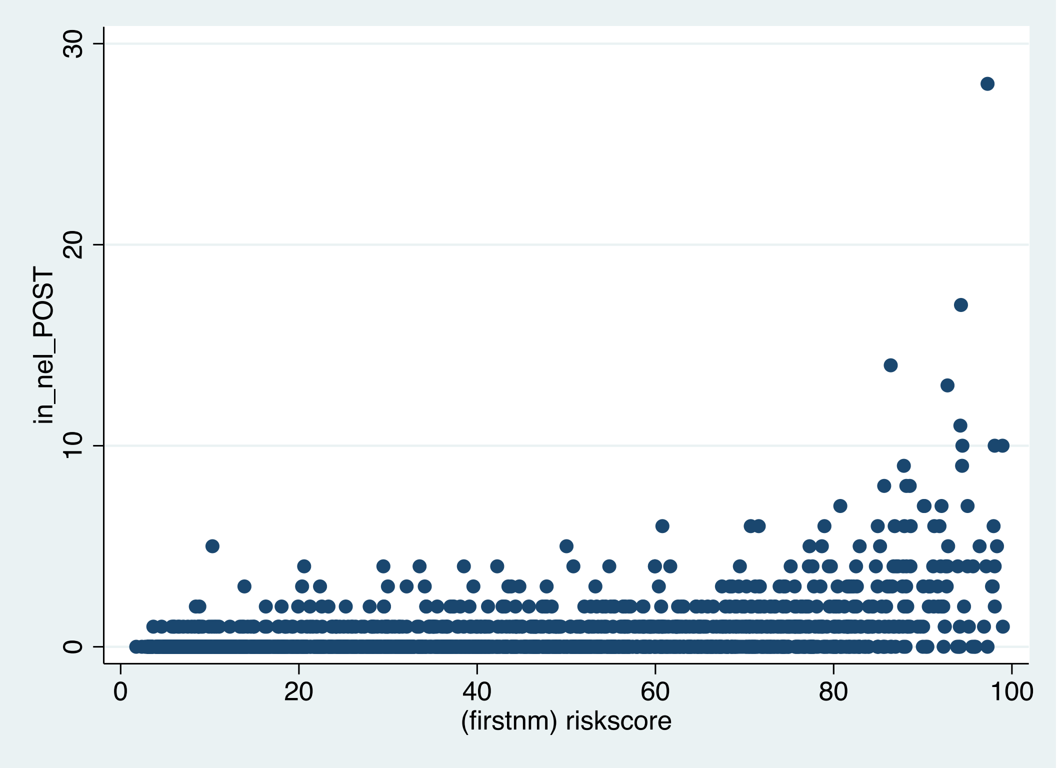

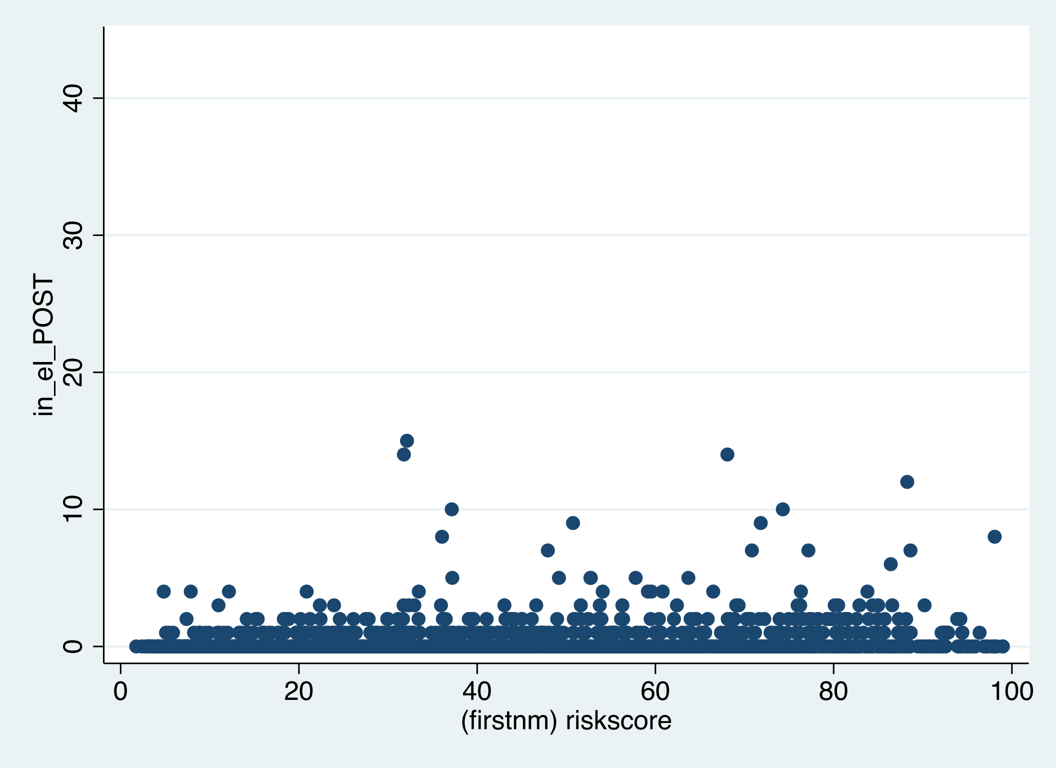
in_el_POST
0
10
20
30
40
0
20
40
60
80
100
(firstnm) riskscore

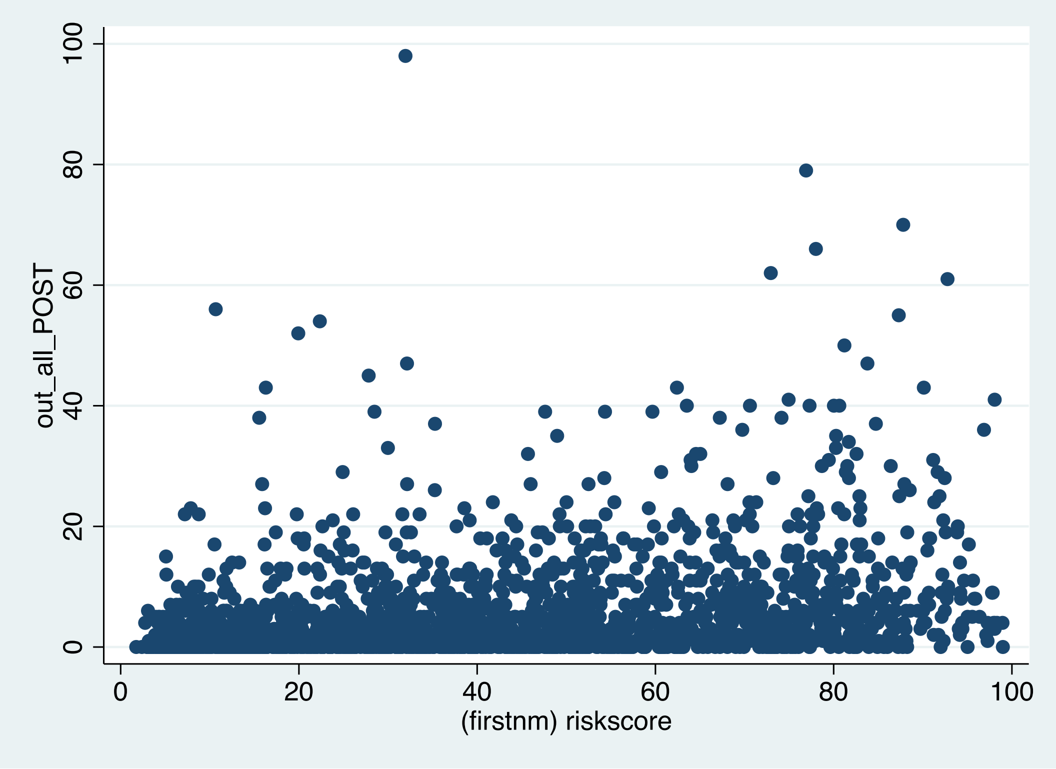
out_all_POST
0
20
40
60
80
100
0
20
40
60
80
100
(firstnm) riskscore

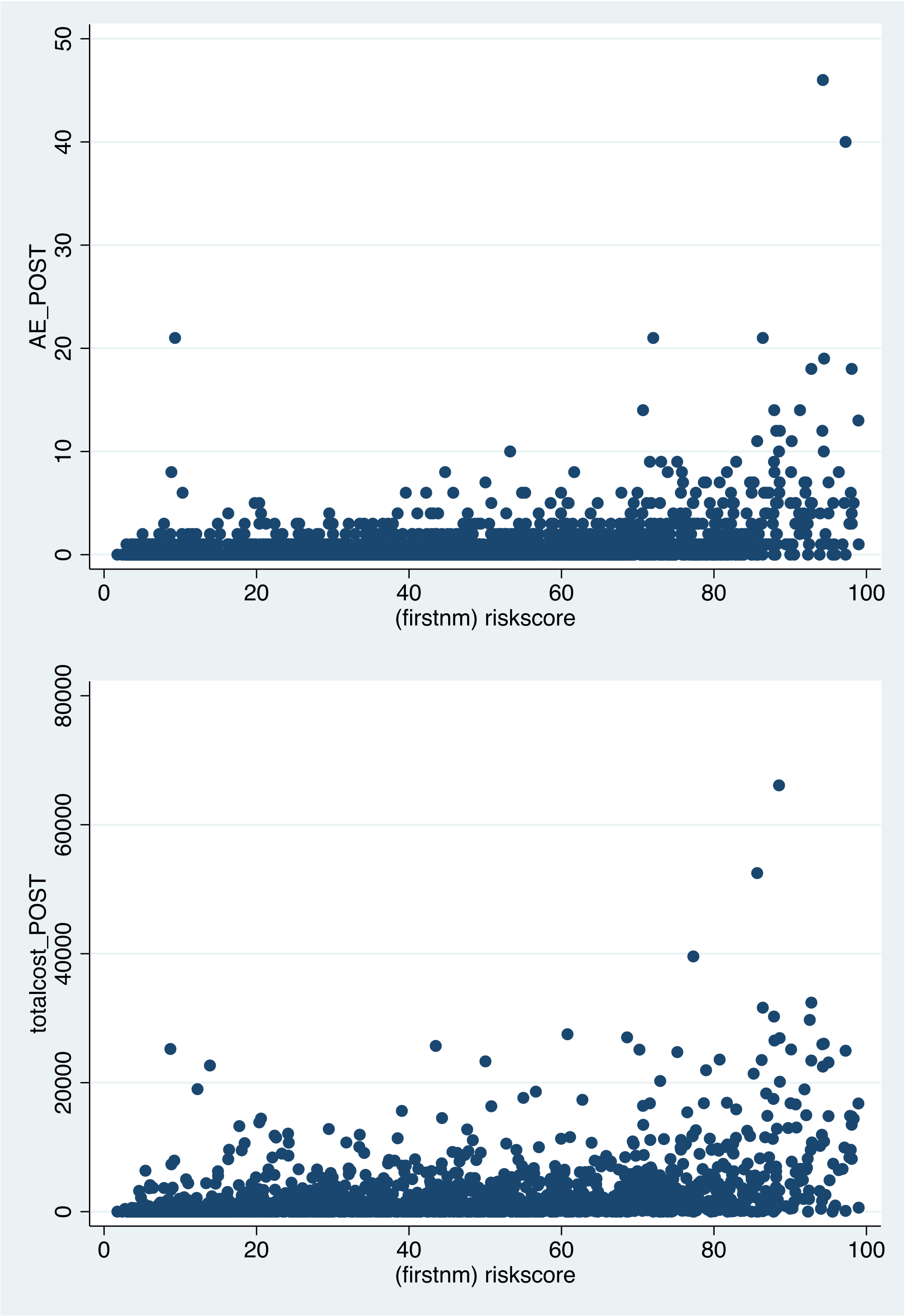
AE_POST
0
10
20
30
40
50
0
20
40
60
80
100
(firstnm) riskscore
totalcost_POST
0
20000
40000
60000
80000
0
20
40
60
80
100
(firstnm) riskscore

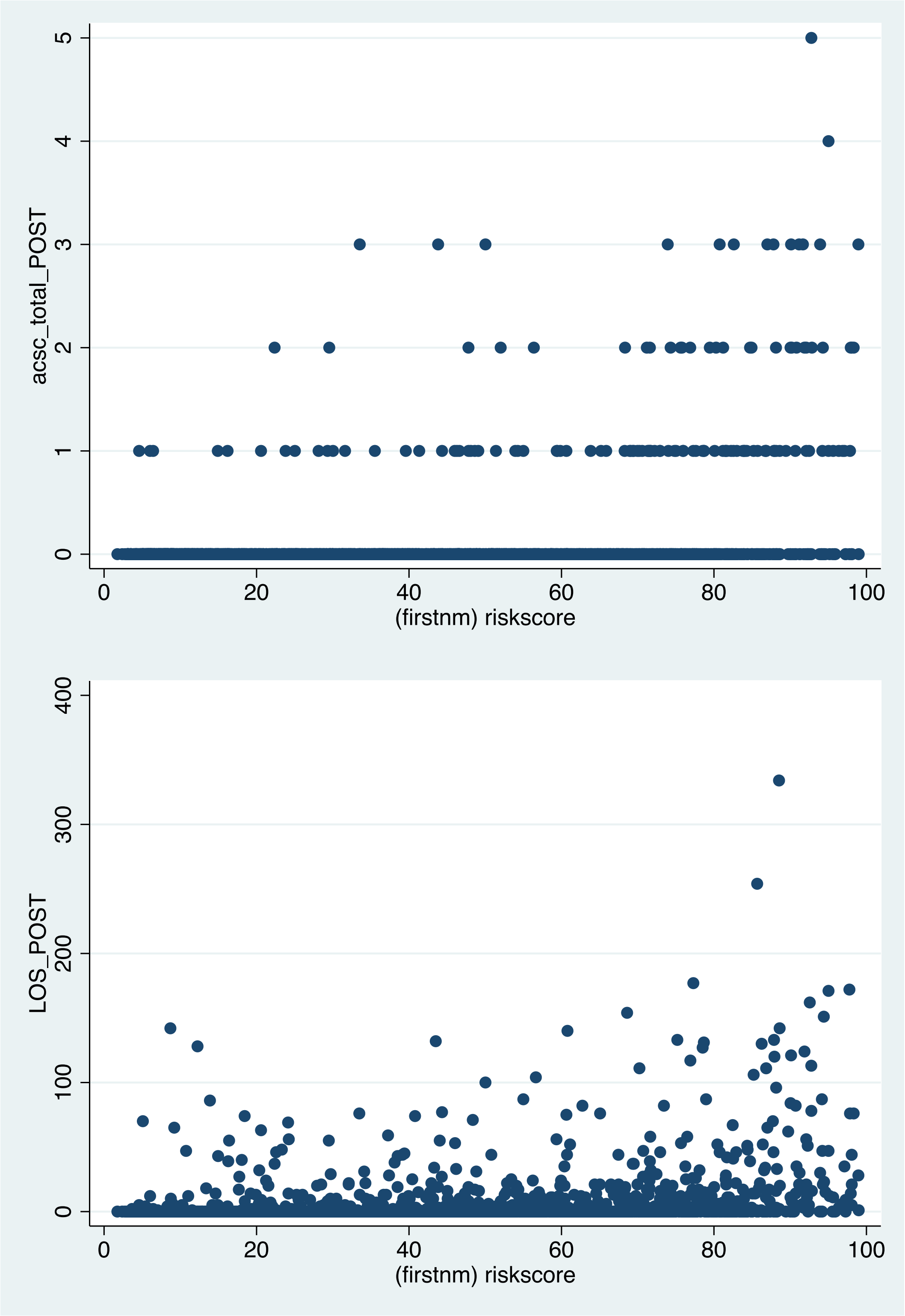
acsc_total_POST
0
1
2
3
4
5
0
20
40
60
80
100
(firstnm) riskscore
LOS_POST
0
100
200
300
400
0
20
40
60
80
100
(firstnm) riskscore

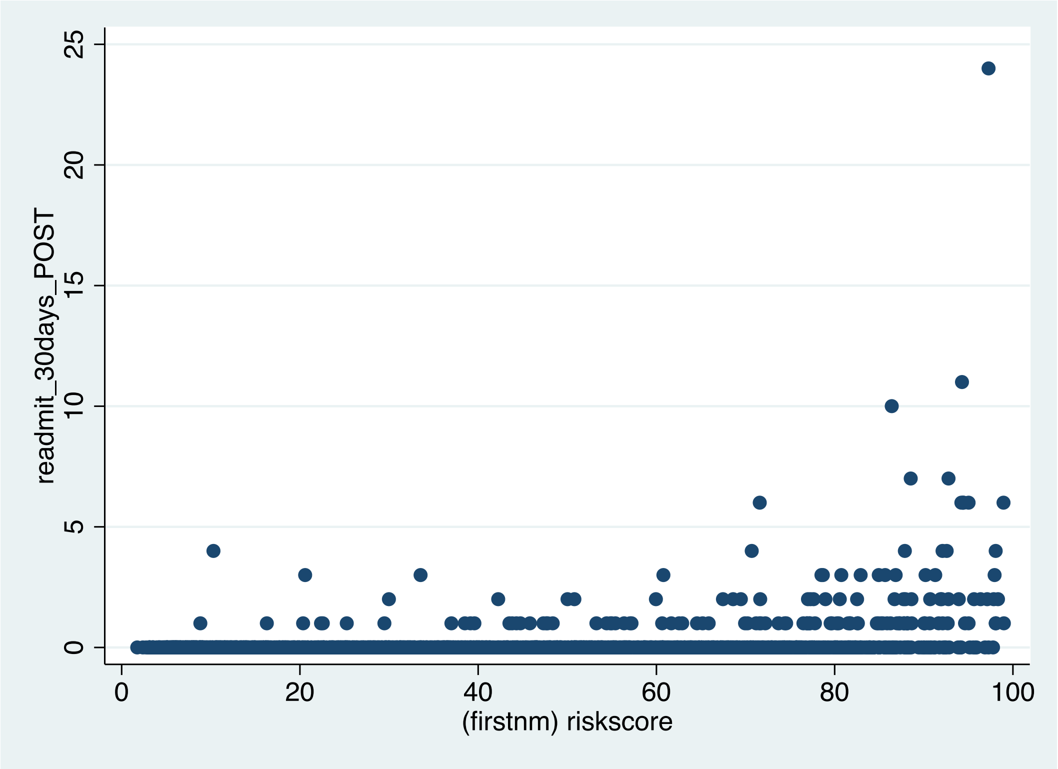

readmit_30days_POST
0
5
10
15
20
25
0
20
40
60
80
100
(firstnm) riskscore

Risk Score DDD Estimates

Outcome	Adjusted+ intervention effect (95% CI) (difference per patient per month)		Effect size*
	**Count (nbreg) model**		
**Primary outcomes:**			
Inpatient non-electives	0.0208 (0.0083 to 0.0333)	#	0.09
Inpatient electives	-0.0009 (-0.0174 to 0.0156)		-0.00
Outpatient admissions	0.0943 (-0.0042 to 0.1927)		0.08
A&E visits	0.0363 (0.0128 to 0.0598)	#	0.09
ACSCs	0.0020 (-0.0029 to 0.0069)		0.02
Re-admissions (30 days)	0.0059 (-0.0004 to 0.0123)		0.05
**Secondary outcomes:**			
Total cost of 2° care services (£)~	19.2166 (-20.5574 to 58.9907)		0.02
Length of stay (days)	0.3071 (0.0592 to 0.5549)	#	0.06

+: Adjusted for: age, cumulative multimorbidity, imd domains (excluding health), practice- and time- fixed-effects. Marginal effects on *PICT x Post* reported.
*: Standardised mean difference
#: significant at p<0.05
nbreg: negative binomial regression
~: zero-inflated negative binomial models based on admission events
n= 224,898 observations; 4098 individuals (period September 2010 to March 2015)

References

1. Wennberg D, Siegel M, Darin B, Filipova N, Russell R, Kenney L, et al. Combined predictive model: final report and technical documentation. *London: Department of Health, The Kings Fund, NYU, Health Dialogue* 2006.
2. Hill J. Reducing bias in treatment effect estimation in observational studies suffering from missing data. 2004.
3. StataCorp. *Stata Statistical Software: Release 13*. College Station, TX: StataCorp LP., 2013.
4. Leuven E, Sianesi B. PSMATCH2: Stata module to perform full Mahalanobis and propensity score matching, common support graphing, and covariate imbalance testing. *Statistical Software Components* 2014.
5. Becker SO, Ichino A. Estimation of average treatment effects based on propensity scores. *The stata journal* 2002;2(4):358-77.
6. Cohen J. *Statistical Power Analysis for the Behavioral Sciences*: L. Erlbaum Associates, 1988.
7. Baines DL, Whynes DK. Selection bias in GP fundholding. *Health economics* 1996;5(2):129-40.
8. Propper C, Croxson B, Shearer A. Waiting times for hospital admissions: the impact of GP fundholding. *Journal of health economics* 2002;21(2):227-52.
9. HSCIC. Quality and Outcomes Framework (QOF) - 2013-14, 2014.
10. Koller D, Schon G, Schafer I, Glaeske G, van den Bussche H, Hansen H. Multimorbidity and long-term care dependency--a five-year follow-up. *BMC geriatrics* 2014;14:70.
11. World Health Organization. International statistical classification of diseases and health related problems (The) ICD-10. World Health Organization, 2004.
12. Tonelli M, Wiebe N, Fortin M, Guthrie B, Hemmelgarn B, James M, et al. Methods for identifying 30 chronic conditions: application to administrative data. *BMC Medical Informatics and Decision Making* 2015;15(1):31.
13. Fortin M, Stewart M, Poitras M-E, Almirall J, Maddocks H. A Systematic Review of Prevalence Studies on Multimorbidity: Toward a More Uniform Methodology. *The Annals of Family Medicine* 2012;10(2):142-51.
14. HSCIC. Numbers of Patients Registered at a GP Practice - January 2014, 2014.
15. StataCorp L. *Stata Time-series: Reference Manual*: StataCorp LP, 2007.
16. Ansari Z, Laditka JN, Laditka SB. Access to health care and hospitalization for ambulatory care sensitive conditions. *Medical care research and review : MCRR* 2006;63(6):719-41.
17. Caminal J, Starfield B, Sanchez E, Casanova C, Morales M. The role of primary care in preventing ambulatory care sensitive conditions. *European journal of public health* 2004;14(3):246-51.
18. Parchman ML, Culler S. Primary care physicians and avoidable hospitalizations. *The Journal of family practice* 1994;39(2):123-8.
19. NHS England. Everyone Counts: Planning for Patients 2013/14 Technical Definitions: NHS Commissioning Board, 2012:1-103.
20. Ipsos MORI. GP Patient Survey – Technical Annex 2013-2014 annual report: Social Research Institute, 2014.

21. Hernan MA, Hernandez-Diaz S, Werler MM, Mitchell AA. Causal knowledge as a prerequisite for confounding evaluation: an application to birth defects epidemiology. *Am J Epidemiol* 2002;155(2):176-84.
